# α‐Synuclein antisense transcript SNCA‐AS1 regulates synapses‐ and aging‐related genes suggesting its implication in Parkinson's disease

**DOI:** 10.1111/acel.13504

**Published:** 2021-11-19

**Authors:** Federica Rey, Cecilia Pandini, Letizia Messa, Rossella Launi, Bianca Barzaghini, Roberta Zangaglia, Manuela Teresa Raimondi, Stella Gagliardi, Cristina Cereda, Gian Vincenzo Zuccotti, Stephana Carelli

**Affiliations:** ^1^ Department of Biomedical and Clinical Sciences "L. Sacco" University of Milan Milan Italy; ^2^ Pediatric Clinical Research Center Fondazione “Romeo ed Enrica Invernizzi” University of Milan Milan Italy; ^3^ Genomic and post‐Genomic Center IRCCS Mondino Foundation Pavia Italy; ^4^ Department of Biology and Biotechnology “L. Spallanzani” University of Pavia Pavia Italy; ^5^ Department of Chemistry, Materials and Chemical Engineering Politecnico di Milano Milan Italy; ^6^ Parkinson's Disease and Movement Disorders Unit IRCCS Mondino Foundation Pavia Italy; ^7^ Department of Pediatrics Children's Hospital "V. Buzzi" Milan Italy; ^8^ Present address: Department of Women, Mothers and Neonatal Care Children's Hospital "V. Buzzi" Milan Italy

**Keywords:** aging, LncRNAs, Parkinson's disease, RNA‐sequencing, SNCA, SNCA‐AS1, synapses, synuclein

## Abstract

SNCA protein product, α‐synuclein, is widely renowned for its role in synaptogenesis and implication in both aging and Parkinson's disease (PD), but research efforts are still needed to elucidate its physiological functions and mechanisms of regulation. In this work, we aim to characterize SNCA‐AS1, antisense transcript to the SNCA gene, and its implications in cellular processes. The overexpression of SNCA‐AS1 upregulates both SNCA and α‐synuclein and, through RNA‐sequencing analysis, we investigated the transcriptomic changes of which both genes are responsible. We highlight how they impact neurites' extension and synapses' biology, through specific molecular signatures. We report a reduced expression of markers associated with synaptic plasticity, and we specifically focus on GABAergic and dopaminergic synapses, for their relevance in aging processes and PD, respectively. A reduction in SNCA‐AS1 expression leads to the opposite effect. As part of this signature is co‐regulated by the two genes, we discriminate between functions elicited by genes specifically altered by SNCA‐AS1 or SNCA's overexpression, observing a relevant role for SNCA‐AS1 in synaptogenesis through a shared molecular signature with SNCA. We also highlight how numerous deregulated pathways are implicated in aging‐related processes, suggesting that SNCA‐AS1 could be a key player in cellular senescence, with implications for aging‐related diseases. Indeed, the upregulation of SNCA‐AS1 leads to alterations in numerous PD‐specific genes, with an impact highly comparable to that of SNCA's upregulation. Our results show that SNCA‐AS1 elicits its cellular functions through the regulation of SNCA, with a specific modulation of synaptogenesis and senescence, presenting implications in PD.

## Graphical abstract

In this work, we aim to characterize SNCA‐AS1, antisense transcript to the SNCA gene, and its implications in cellular processes. Through RNA‐sequencing, we investigated the transcriptomic changes of which both genes are responsible, highlighting a role for them in Parkinson's disease, synapses biology, and aging processes.

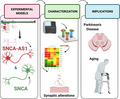

**Abbreviations**
α‐syn: α‐synuclein
BP: biological process
C2: curated gene set
C5: ontology gene set
CC: cellular component
DE RNAs: differentially expressed RNAs
EV: empty vector
GO: gene ontology
GSEA: gene set enrichment analysis
iPSCs: induced pluripotent stem cells
KEGG: kyoto encyclopedia of genes and genomes
lncRNA: long non‐coding RNA
MF: molecular function
MFE: minimum free energy
ML: maximum‐likelihood
NJ: neighbor‐joining
PBMCs: peripheral blood mononuclear cells
PD: Parkinson’s disease
RNA‐Seq: RNA‐sequencing
SNCA‐AS1: SNCA antisense gene
SNCA: synuclein alpha gene
SNP: single nucleotide polymorphisms
TF: transcription factor
TUJ: beta‐TubIII
VMAT: vesicular monoamine transporter

## 1 INTRODUCTION

Aging is the main risk factor for Parkinson's disease (PD), whose prevalence increases by more than 400 times in the elderly population reaching 1% at the age of 60 and 5% in people over 85 (Rodriguez et al., 2015). Numerous neurodegenerative processes occurring in PD are linked to the aging brain, with a great relevance for the degeneration of the nigrostriatal dopaminergic neurons system, a crucial hallmark of PD. Indeed, it has been reported that the most significant difference in PD and aged brains is the number of dopaminergic neurons, suggesting that the etiology of the disease could be overlapping with aging (Rodriguez et al., 2015). The synuclein alpha gene (*SNCA*) encodes for α‐synuclein (α‐syn), the small protein mainly known for its implications in PD (Houlden & Singleton, 2012) and principal component of the Lewy Bodies aggregates (Spillantini et al., 1997), the second fundamental hallmark of PD (Poewe et al., 2017). α‐syn physiological functions are still far from clarified. Molecular and transgenic studies involving the protein have not yet completely elucidated its functions, although its main implication seems to be in the synaptic vesicles cycle and dendritic development (Sulzer & Edwards, 2019). In both the aging brain and PD, α‐syn promotes the disruption of synapses, with the accumulation of pathogenic proteins leading to an impairment of the ubiquitin‐proteasome system, autophagy, and mitochondria, ultimately resulting in dopaminergic degeneration (Bobela et al., 2015; Rodriguez et al., 2015).

It is necessary to eviscerate the possible implication of the *SNCA* gene locus in α‐syn's regulation, as it could represent a common target in PD and aging. Epigenetically, *SNCA* is highly modulated through twenty‐one CpG islands present on the promoter and the first intron of the gene (Guhathakurta et al., 2017). In addition, the SNCA antisense gene (SNCA‐AS1), which codes for a long non‐coding RNA (lncRNA), has been recently described to localize on the strand opposite to the *SNCA* gene (Fagerberg et al., 2014). Indeed, the SNCA locus, including SNCA‐AS1, has been recently associated with hereditary neurodegenerative diseases and Lewy body dementia (Chia et al., 2021; Zucchelli et al., 2019). Conversely to SNCA's mRNA, SNCA‐AS1 is strongly enriched in brain tissue (Fagerberg et al., 2014), and its expression increases during both SH‐SY5Y cells and induced Pluripotent Stem Cells (iPSCs) dopaminergic in vitro differentiation (Elkouris et al., 2019). There is currently no evidence reported of what its role in SNCA's regulation and in both aging and PD pathogenesis might be, nor a clear understanding of its function at a cellular level. A wide range of lncRNAs are being implicated in aging and neurodegenerative diseases, through modulation of autophagy, apoptosis, oxidative stress, and even SNCA's expression and subsequent effects on α‐syn's aggregation (Lyu et al., 2019; Pereira Fernandes et al., 2018). LncRNAs could even play a role as potential biomarkers, as a significant number of them have been found deregulated in circulating leucocytes or peripheral blood mononuclear cells (PBMCs) from sporadic PD patients (Fan et al., 2019).

It is in this context that there is a crucial need to understand the potential implication for SNCA‐AS1 in SNCA's regulation, as it could provide new therapeutic strategies and it could also elucidate new functions underlying cellular senescence and ultimately neurodegenerative diseases. With this work, we aim to elucidate the effect of SNCA‐AS1 and SNCA mRNA overexpression in the in vitro SH‐SY5Y cell line, a neural model consolidated for its relevance in PD studies (Xicoy et al., 2017). We report how the overexpression of SNCA‐AS1 leads to an increase in SNCA's mRNA expression, and through RNA sequencing, we identify a significant number of deregulated genes specifically ascribable to SNCA‐AS1 or SNCA's mRNA overexpression. With both bioinformatic approaches and in vitro validations, we describe how these genes influence numerous processes, including neurites extension, synaptogenesis, and cellular senescence, with significant implications in the brain aging process and in PD pathology.

## 2 RESULTS

### 2.1 In silico characterization of the novel SNCA Antisense RNA SNCA‐AS1

**FIGURE 1:**
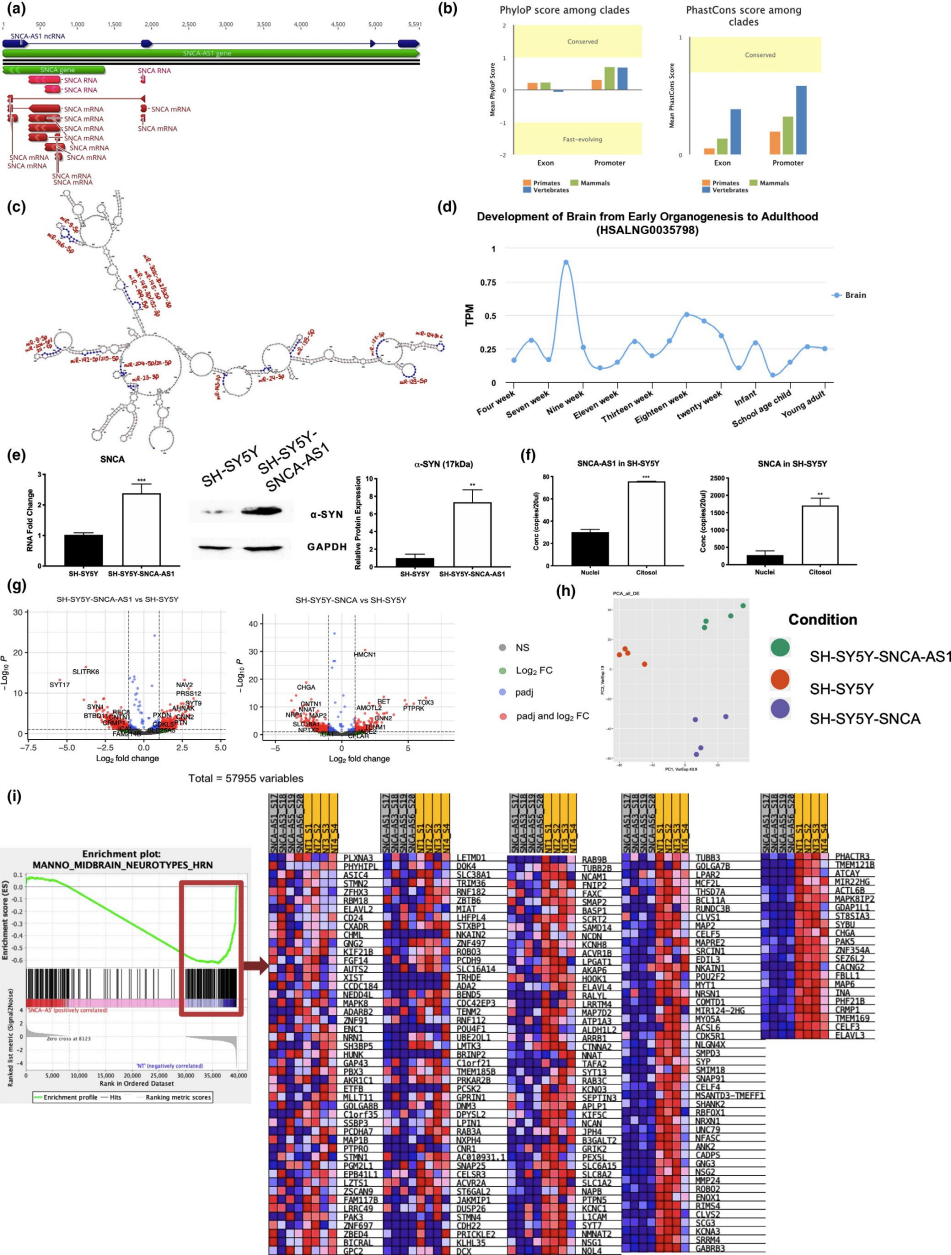
α‐synuclein's (α‐syn) expression can be modulated by SNCA‐AS1 and effects of both SNCA‐AS1 and SNCA overexpression on transcription profiles. (a) Genetic localization of SNCA‐AS1 gene with respect to the SNCA gene. (b) Evolution conservation as obtained with the AnnoLnc2 database. (c) SNCA‐AS1 RNA secondary structure prediction for minimum free energy (MFE) minimization with the AnnoLnc2 database. Binding sites for miRNA are highlighted in blue, and the respective miRNA in red. (d) SNCA‐AS1 expression in the brain as obtained with the LncExpDB database. (e) The increase of α‐syn's expression in SH‐SY5Y‐SNCA‐AS1 was evaluated by means of real‐time PCR and Western blot. GAPDH was used as housekeeping gene for both experiments. For real‐time PCR analysis, data are expressed as mean ± SEM of 3 replicate values in 3 independent experiments (n = 9; ***p < 0.001 vs. SH‐SY5Y). For Western blot analysis, data are expressed as mean ± SEM of 5 independent experiments (n = 5; **p < 0.01 vs. SH‐SY5Y). (f) Cellular localization determined by droplet digital PCR in SH‐SY5Y. Data are expressed as mean ± SEM of 3 independent experiments (n = 3; **p < 0.01, ***p < 0.001 vs. Nuclei). (g) Volcano plot showing deregulated genes in SH‐SY5Y‐SNCA‐AS1 vs SH‐SY5Y and SH‐SY5Y‐SNCA vs SH‐SY5Y. (h) PCA of differently expressed genes in SH‐SY5Y‐SNCA‐AS1, SH‐SY5Y‐SNCA, and SH‐SY5Y. (i) GSEA representation of MANNO midbrain neurotype as obtained with the cell type signature gene sets database

SNCA antisense RNA (SNCA‐AS1) is a recently discovered antisense transcript which localizes on chromosome 4, on the strand opposite to the *SNCA* gene, partially overlapping its 5′ end with its first exon (Figure 1a). Specifically, its second exon overlaps the first exon of the longest isoform of the SNCA mRNA. With the AnnoLnc2 database, we screened the lncRNA for repeat elements, evolutionary conservation, transcriptional, and miRNA regulation (Ke et al., 2020). The search for repeat elements with the RepeatMasker genomic datasets highlighted no results, suggesting these elements are not present along SNCA‐AS1 sequence. PhyloP and PhastCons scoring aimed at assessing base conservation highlighted a tendency for sequence conservation across primates, mammals, and vertebrates (Figure 1b). Moreover, phylogenetic analysis was performed by neighbor‐joining (NJ) method and the maximum‐likelihood (ML) based on 26 aligned predicted sequences of SNCA‐AS1 (see Experimental Procedures). *Homo sapiens* was used as outgroup. The NJ‐based phylogenetic analysis allows to distinguish between four groups, reporting a conservation of the transcripts in mammals. 19 out of 26 are primates, which are mostly grouped together with exclusion of the *Cercopithecidae familiae* (Figure S1A). Phylogenetic analysis highlights also similarity between predicted sequences with *Camelus*, *Vicugna*, and *Mustela* (Figure S1A). The ML‐based phylogenetic tree shows similar topology with the NJ‐based phylogenetic tree (Figure S1B). SNCA‐AS1's secondary structure was predicted through conformational bioinformatics studies with the identification of a possible secondary structure specifically based on the minimum free energy (MFE) minimization (Figure S1C), predicted according to Turner 2004 RNA folding parameters (Lorenz et al., 2011). The MFE reached is −244.30 kcal/mol. We investigated the potential miRNA regulation of SNCA‐AS1 through the analysis of miRNA binding sites and we highlighted binding motifs for 17 miRNA (Figure 1c, Table S1). Transcription factors (TFs) associated with SNCA‐AS1 were predicted using both the AnnoLnc2 database through the Gene Transcription Regulation Database database (Table S2) and Ciiider software (Gearing et al., 2019), which highlighted 462 TFs. Among them, 21 out of 462 were neural TFs (Figure S1D) and, even more interestingly, 9 (e.g., Ahr::Arnt, Arnt, E2f1, Foxj2, Foxo1, Foxo4, Hsf1, Sox5, and Yy1) were associated with the aging process (Figure S1E). An expression analysis conducted with the LncExpDB (Li et al., 2021) database highlighted a strong expression of SNCA‐AS1 during brain development, with a peak at week 8 (Figure 1d).

### 2.2 SNCA‐AS1 regulates SNCA and α‐synuclein's expression

To firstly investigate the possible regulation of SNCA by SNCA‐AS1, we analyzed whether this lncRNA could impact SNCA's transcription and subsequent translation. Indeed, SH‐SY5Y cells stably transfected with SNCA‐AS1 presented an upregulation of SNCA's mRNA expression and a subsequent upregulation of its protein product α‐syn (Figure 1e). As very little is known about SNCA‐AS1's expression and function, an analysis of its localization was performed by droplet digital PCR. The results show that SNCA‐AS1 and SNCA RNAs are mainly localized in the cytoplasm, both in non‐transfected SH‐SY5Y (Figure 1f) and when overexpressed (SH‐SY5Y‐SNCA and SH‐SY5Y‐SNCA‐AS1) (Figure S1F).

### 2.3 Overexpression of SNCA‐AS1 and SNCA leads to significantly different RNAs expression profiles

As SNCA‐AS1 is a novel uncharacterized lncRNAs, nothing is known about its signal transduction and there is no evidence of specific cellular pathways affected by its expression. Furthermore, the effect of SNCA's mRNA overexpression on cellular transcriptome is yet to be fully characterized and could be helpful in the understanding of α‐syn's biology and PD pathogenesis. To this end, a whole transcriptome analysis of wild type SH‐SY5Y and SH‐SY5Y overexpressing either SNCA‐AS1 or SNCA was performed. We detected many differentially expressed coding and non‐coding RNAs (DE RNAs) in SH‐SY5Y‐SNCA‐AS1 and SH‐SY5Y‐SNCA vs. SH‐SY5Y, as shown by volcano plot analysis (Figure 1g). PCA analysis of the DE RNAs showed a clear difference in the three expression profiles, suggesting that the two investigated genes deeply affect cellular functions (Figure 1h). Specifically, a total of 969 transcripts were affected by SNCA‐AS1's overexpression (82% of which were coding genes, Table S3) and 698 by SNCA's overexpression (85% of which were coding genes, Table S4). A validation of 4 selected deregulated transcripts, implicated in pathways of interest, was performed by real‐time PCR confirming the RNA‐Sequencing (RNA‐Seq) evidence (Figure S2). Gene set enrichment analysis (GSEA) for the cell type signature gene sets database was performed, and, interestingly, we found that among the top cellular phenotypes impacted by SNCA‐AS1, the midbrain neurotype appeared to be profoundly negatively correlated with the lncRNA overexpression, with a high number of genes being downregulated (Figure 1i).

### 2.4 Pathway analysis of deregulated transcripts

**FIGURE 2:**
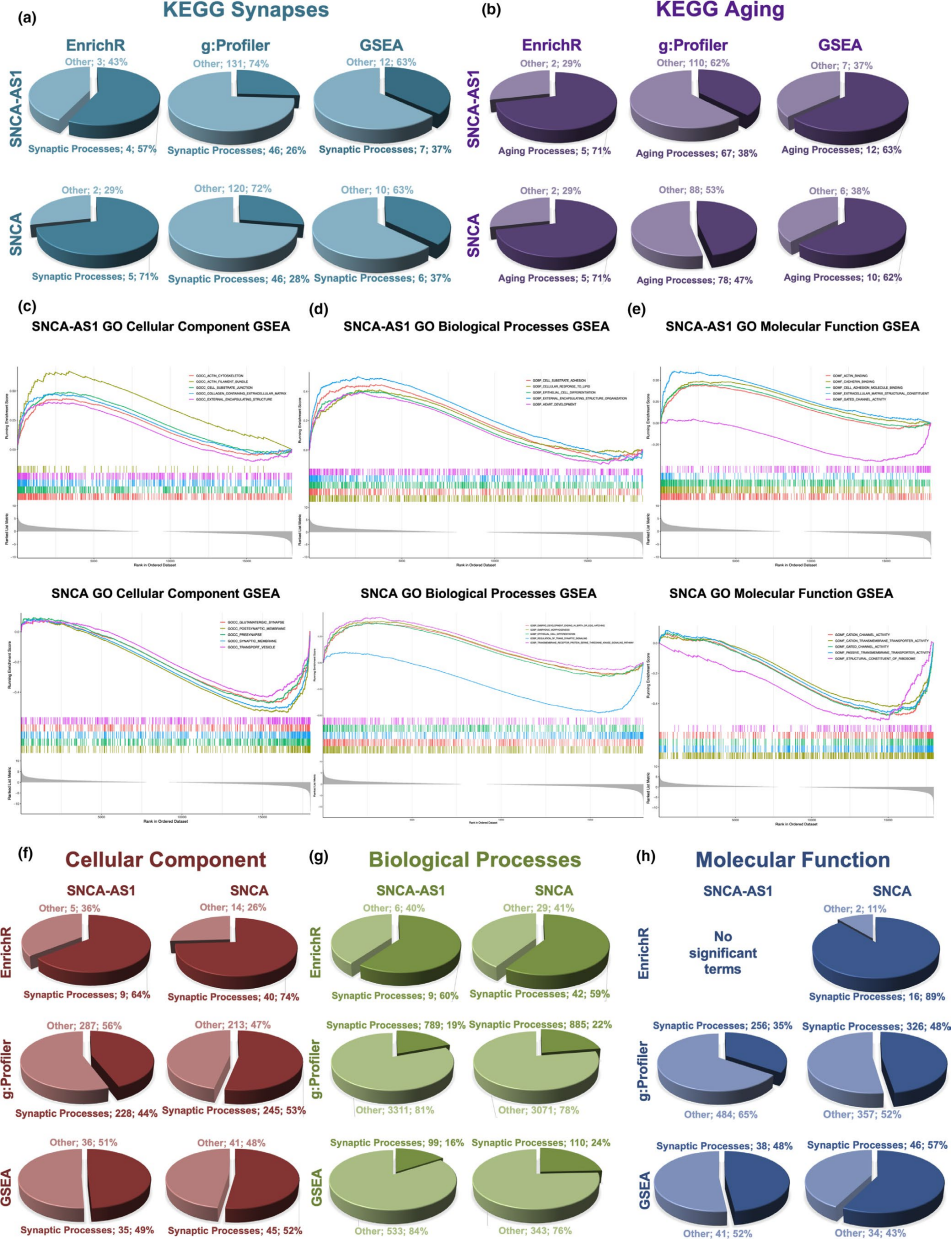
Pathway and GO analyses for DE RNAs in SH‐SY5Y overexpressing SNCA‐AS1 and SNCA. Pie chart displaying the % of KEGG processes associated with synapses (a) or aging (b) processes as obtained with EnrichR, g:Profiler and GSEA analyses in SH‐SY5Y‐SNCA‐AS1 or SH‐SY5Y‐SNCA vs. SH‐SY5Y. Only terms with *p*‐value <0.05 were considered. GSEA analysis of top GO terms for CC (c), BP (d), and MF (e) in SH‐SY5Y‐SNCA‐AS1 or SH‐SY5Y‐SNCA vs SH‐SY5Y. Pie chart displaying the % of processes associated with synapses in GO CC (f) BP (g) or MF (h) as obtained with EnrichR, g:Profiler, and GSEA analyses in SH‐SNCA‐AS1 or SNCA vs SH‐SY5Y. Only terms with *p*‐value <0.05 were considered

Transcripts were subjected to Kyoto Encyclopedia of Genes and Genomes (KEGG) enrichment analysis using GSEA (Table S5), and in Figure S3, the significantly enriched terms obtained with GSEA KEGG pathway analysis are reported. Interestingly, in SNCA overexpression it is possible to notice a reduced expression of terms associated with neurodegenerative diseases (Alzheimer's disease and PD) (Figure S3). The deregulated transcripts with a deregulation ≥1 in terms of |Log_2_FC| were subjected to KEGG pathways analysis through the use of two more tools: the EnrichR web tool (Kuleshov et al., 2016) (Table S6) and g:Profiler (Table S7), where terms were ranked for their importance in terms of fold change (Raudvere et al., 2019). As the only evidence currently present for SNCA‐AS1 points to its implication in neural differentiation (Elkouris et al., 2019), along with a synaptic implication being the main physiological function known for SNCA, we extrapolated from the three enrichment analysis datasets all pathways pertaining synaptogenesis (Figure 2a). All three enrichment methods highlighted that around 1/3 of terms relate to synaptogenesis in both SNCA‐AS1 and SNCA‐overexpressing cells (the only exception being the EnrichR analysis, where for SH‐SY5Y‐SNCA‐AS1 57% of reported pathways and for SH‐SY5Y‐SNCA 71% of reported pathways is connected to synaptogenesis). Moreover, as aging‐associated diseases appeared among the top enriched pathways with GSEA analysis, and as SNCA has been found implicated in physiological aging, we also highlighted pathways pertaining aging processes (Figure 2b). A relevant number of pathways resulted associated for both conditions with aging processes in all three used databases, with EnrichR and GSEA showing more than 60% associated terms, g:Profiler around 40% for both conditions (Figure 2b).

### 2.5 Gene ontology profiles predict specific functions for SNCA‐AS1 and SNCA

Gene expression profiles of SH‐SY5Y‐SNCA‐AS1 and SH‐SY5Y‐SNCA were analyzed for gene ontology (GO) cellular component (CC) enrichment via GSEA, to identify the possible phenotypic alteration induced by the two genes' overexpression (Figure S4A,B). Interestingly, out of the top 20 pathways ranked for their *p*‐value obtained for SNCA‐AS1 16 indicate a positive enrichment score, and a high number of these pertained complexes associated with cytoskeletal remodeling (e.g., actin, extracellular matrix and basement membrane, with the four negatively enriched ones related to synaptic processes [Figure S4A]). On the contrary, 18 out of 20 pathways for SNCA presented a negative enrichment and pertained synaptic processes such as transporter complexes, cation channel complex, post‐synaptic membrane and more (Figure S4B). We also performed a GO analysis for CC, biological processes (BP), and molecular function (MF) in both datasets (Figure 2c‐e, Tables [Link], [Link], [Link]). The top 5 deregulated processes for GO CC in SNCA‐AS1‐overexpressing cells highlighted a positive enrichment in processes involved in cytoskeleton, cell‐substrate junction, and extracellular matrix remodeling (Figure 2c). SNCA overexpression leads to a profound decrease in enrichment for pathways associated with synaptic transmission processes such as glutamatergic synapse, post‐synaptic membrane, pre‐synapse, and synaptic membrane (Figure 2c). When considering the GO BP, it can be noted that the top 5 deregulated processes affected by the overexpression for SNCA‐AS1 all present a positive enrichment related to heart development, cell and epithelial differentiation, cell‐substrate adhesion, and cellular response to lipids (Figure 2d). For SNCA, BP pathways present both a positive enrichment, related to embryonic development and transmembrane receptor protein serine threonine kinase signaling, and a negative enrichment related to trans‐synaptic signaling (Figure 2d). Lastly, we examined GO MF, finding that 4/5 terms presented positive enrichment in SNCA‐AS1 pathways, primarily related to cell morphology re‐arrangements, while the negatively enriched one was related to gated channel activity (Figure 2e). On the contrary, the enrichment for SNCA GO MF consisted of negative values, related to neurotransmitter activity, cation channels activity, and ribosomes (Figure 2e).

In order to obtain more information on SNCA‐AS1 involvement in regulating synaptic processes, as it can be relevant for both PD and aging progression, the GO CC, BP, and MF enrichment were analyzed using GSEA (Table S8), EnrichR (Table S9), and g:Profiler (Table S10). When considering the CC profile, all three enrichment methods reported a strong involvement of both SNCA and SNCA‐AS1 in synaptic processes, which appears to be around 50% for most databases analysis (except for EnrichR where it seems to comprise most processes) (Figure 2f). Considering BP, the synaptic involvement ranged from 15% to 25% for g:Profiler and GSEA analysis, reaching 60% with the EnrichR database (Figure 2g). Lastly, for MF analysis, the synaptic implication ranged from 35%–57% for g:Profiler and GSEA analysis, reaching 89% with the EnrichR database (Figure 2h).

### 2.6 SNCA‐AS1 and SNCA transcriptional modifications are implicated in synaptic modulation resembling an aging‐related decline

**FIGURE 3:**
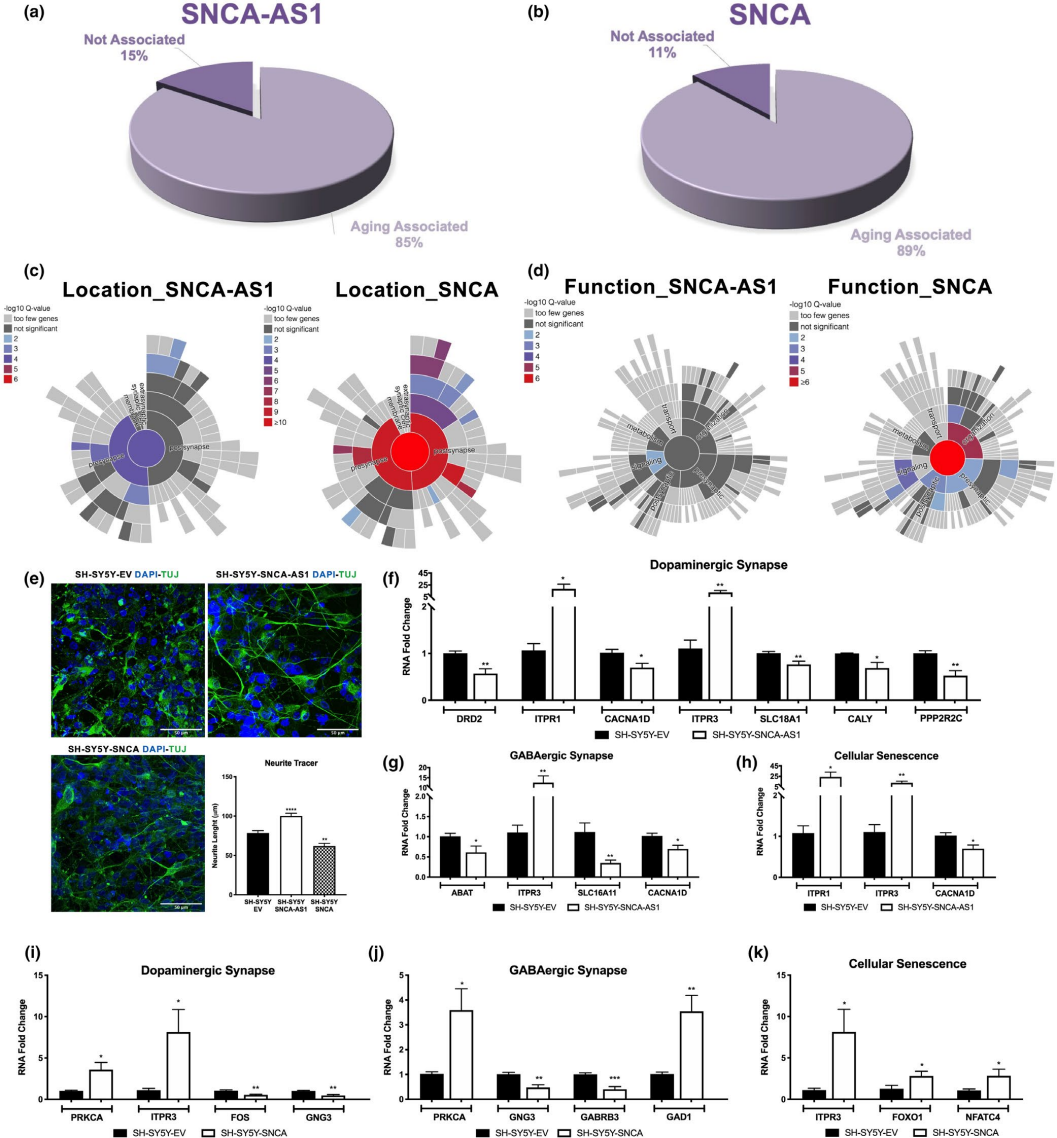
Involvement for SNCA‐AS1 and SNCA in synaptogenesis. Pie chart displaying the amount (percentage) of processes associated with aging as obtained with the Aging Atlas database for SNCA‐AS1 (a) or SNCA (b). Sunburst plot of genes with a synaptic location (c) or functions (d), respectively, as obtained with the SynGO database. (e) Representative immunofluorescence images of SH‐SY5Y‐EV, SH‐SY5Y‐SNCA‐AS1, and SH‐SY5Y‐SNCA labeled for TUJ. Nuclei were labeled in blue (DAPI). Scale bar 50 μm. Neurite Tracer analysis is presented as mean of the quantification of 10 cells performed in 3 different fields for each condition, in 3 independent experiments ±SD (n = 90, **p < 0.01, ****p < 0.0001 vs. SH‐ SY5Y‐EV). Real‐time PCR validation of genes correlated with Dopaminergic Synapse (f), GABAergic Synapse (g), and Cellular Senescence (h) in SH‐SY5Y‐SNCA‐AS1 vs. SH‐SY5Y‐EV. Results are an average of 4 independent experiments performed in duplicates (N = 8, *p < 0.05, **p < 0.01 vs. SH‐SY5Y‐SY5Y‐EV). Real‐time PCR validation of genes correlated with Dopaminergic Synapse (i), GABAergic Synapse (j), and Cellular Senescence (k) in SH‐SY5Y‐SNCA vs. SH‐SY5Y‐EV. Results are an average of 4 independent experiments performed in duplicates (N = 8, *p < 0.05, **p < 0.01 vs. SH‐SY5Y‐EV)

Synaptic dysfunctions are underlying processes in both aging and neurodegenerative diseases, and SNCA‐AS1 could prove to be of crucial relevance. We firstly used the Aging Atlas database to identify those genes, among the DE RNAs, which have been implicated in aging processes through transcriptomics, proteomics, epigenomics, and more studies (Consortium, 2021). We highlighted how in both cases more than 85% of genes are correlated with aging, specifically 85% for SH‐SY5Y‐SNCA‐AS1 (Figure 3a, Table S11) and 89% for SH‐SY5Y‐SNCA (Figure 3b, Table S11). To obtain a comprehensive ontology of synaptic processes and locations, the expression profiles of both SH‐SY5Y‐SNCA‐AS1 and SH‐SY5Y‐SNCA were analyzed using the SynGO database and then visualized as sunburst plot (Koopmans et al., 2019) (Figure 3c,d). Specifically, we found that 99/969 (10.2%) DE RNAs in SH‐SY5Y‐SNCA‐AS1 and 115/698 (16.5%) DE RNAs in SH‐SY5Y‐SNCA were synapses‐involved genes. Indeed, a significant number of genes are reported to be localized at the synapse (both in the pre‐ and post‐synapse) for SNCA, and this is also true for SNCA‐AS1, although to a relatively smaller degree (with a higher implication for the pre‐synapse) (Figure 3c). When considering the genes' functions, it appears that the most significant involvement for SNCA is in the organization process, with a −log10 Q‐value = 5, while for SNCA‐AS1 the main involvement seems to be in signaling, with a −log10 Q‐value = 2 (Figure 3d).

To investigate the possible effect of both SNCA‐AS1 and SNCA on the neuronal phenotype, we analyzed the gene's effect on neurites extension in vitro labeling SH‐SY5Y‐SNCA‐AS1, SH‐SY5Y‐SNCA, and SH‐SY5Y transfected with an empty vector (SH‐EV) and differentiated for 7 days with retinoic acid through the analysis of the neuron‐specific microtubules marker beta‐TubIII (TUJ) (Figure 3e). We found a clear evidence supporting the implication of both SNCA and SNCA‐AS1 in neurites projection. Specifically, these results indicate that the overexpression of SNCA‐AS1 lead to the elongation of neurites, whereas SNCA's overexpression correlates with the opposite (Figure 3e).

To thoroughly investigate the involvement of SNCA‐AS1 in synaptic processes, the deregulated pathways correlated with synaptic enrichment in SH‐SY5Y‐SNCA‐AS1 or SH‐SY5Y‐SNCA were analyzed. We investigated the role of both SNCA‐AS1 and SNCA in regulating the expression of dopaminergic synapse‐related genes, strongly related to both PD pathogenesis and aging process. To do this, a Pathview analysis of dopaminergic synapse pathway in SH‐SY5Y‐SNCA‐AS1 (Figure S5A) and SH‐SY5Y‐SNCA (Figure S5B) was performed, highlighting that in both conditions there seems to be a reduced synaptic plasticity, as highlighted by the decrease in c‐fos, a gene correlated to increased long‐term synaptic plasticity and, intriguingly, implicated in aging (Jaworski et al., 2018). Moreover, in both contexts, there is a decrease in the expression of the gene that codes for vesicular monoamine transporter (VMAT), also decreased in aging neurons (Meyza et al., 2007). A closer look was also given to the impact of both SNCA‐AS1 and SNCA on GABAergic synapses (Figure S6A,B) as these are widely deregulated in the aging process for their relevance in regulating the excitatory/inhibitory balance. Indeed, a significant disruption is observed both in SH‐SY5Y‐SNCA‐AS1 and in SH‐SY5Y‐SNCA with a decreased uptake of synaptic GABA (Figure S6). Lastly, focusing on cellular senescence pathways, both in SH‐SY5Y‐SNCA‐AS1 and SH‐SY5Y‐SNCA (Figure S7A,B) there was an upregulation in related genes, including the FOXO1 transcription factor, whose prediction site is also present in SNCA‐AS1's promoter (Figure S1E) and which is strongly implicated in aging‐related processes (Tia et al., 2018). We validated the above results via real‐time PCR in differentiated SH‐SY5Y‐EV, SH‐SY5Y‐SNCA‐AS1, and SH‐SY5Y‐SNCA. We confirmed the perturbations found in SH‐SY5Y‐SNCA‐AS1 for the dopaminergic synapse (Figure 3f), the GABAergic synapse (Figure 3g), and cellular senescence implicated genes (Figure 3h). Similarly, we validated the alterations found in SH‐SY5Y‐SNCA for the dopaminergic synapse (Figure 3i), the GABAergic synapse (Figure 3j), and cellular senescence implicated genes (Figure 3k).

### 2.7 Investigation of the overlapping and divergent sense/antisense pathways

It is possible to identify from the data presented a clear overlapping in shared processes when either SNCA‐AS1 or SNCA genes are overexpressed. These include synapses‐ and aging‐related pathways, and it could possibly be due to the fact that SNCA‐AS1 overexpression increases SNCA's and subsequently α‐syn's expression and probable functions. It is thus necessary to identify the functions which are solely due to SNCA‐AS1's overexpression, the ones shared among the two and due to a possible inter‐regulation between the molecules and the ones solely due to SNCA's overexpression, in order to potentially target selective aspects of their target transductions. In order to identify specific functions, GO and KEGG analyses were then performed for the three gene sets: 724 genes deregulated only in SH‐SY5Y‐SNCA‐AS1, 245 genes shared among the two conditions, and 453 genes deregulated only in SH‐SY5Y‐SNCA.

**FIGURE 4:**
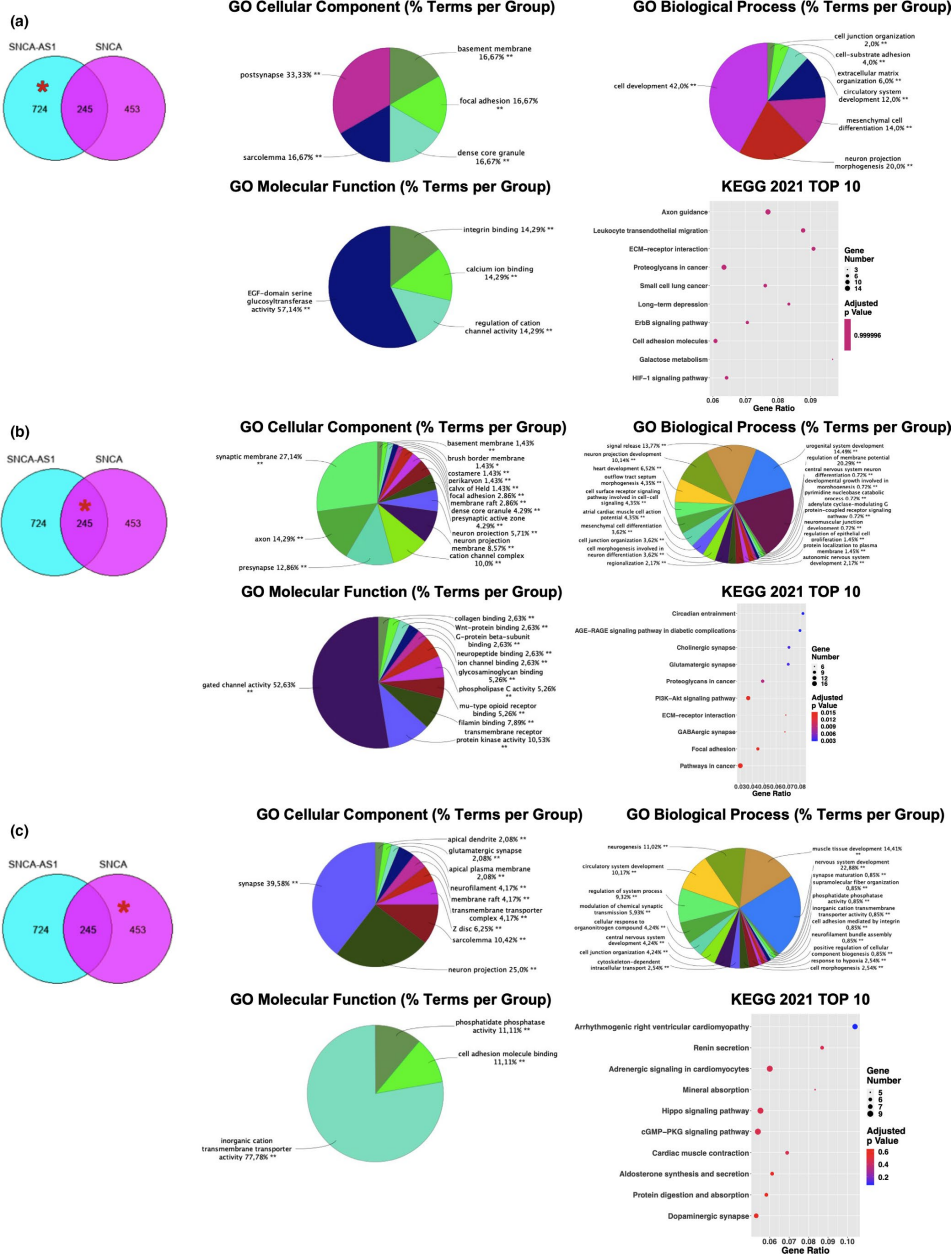
Analysis of SNCA‐AS1 and SNCA‐specific functions. ClueGO CC, MF, BP, and dotplot of KEGG top 10 enriched terms in genes deregulated only SH‐SY5Y‐SNCA‐AS1 vs SH‐SY5Y (a), in genes shared between SH‐SY5Y‐SNCA‐AS1 and SH‐SY5Y‐SNCA vs SH‐SY5Y (b) and in genes deregulated only in SH‐SY5Y‐SNCA vs SH‐SY5Y (c). Each pie segment refers to the % of terms present per group, *p* < 0.05 was set as the limit of significance (**p < 0.01 vs. SH‐SY5Y). In the dotplots, the *y*‐axis represents the name of the pathway, the *x*‐axis represents the gene ratio, dot size represents the number of DE RNAs and the color indicates the adjusted *p*‐value

When the SH‐SY5Y‐SNCA‐AS1 specific genes were analyzed (Figure 4a), the CC analysis demonstrates the most significant enrichment in the post‐synapse compartment, followed by dense core granule, focal adhesion, and basement membrane compartments. Furthermore, the MF indicates a main role in glucosyltransferase activity but also an implication for calcium ion binding and cation channel activity. In the GO BP analysis, there is a main enrichment in cell development, followed by neuron projection morphogenesis. With KEGG 2021 analysis, among the top 10 deregulated pathways it is possible to notice axon guidance, ECM‐receptor interactions, and cell adhesion molecules, along with long‐term depression.

When analyzing GO and pathway enrichment for the 245 shared terms, a more significant synaptic involvement was present (Figure 4b). The GO CC enrichment suggests an alteration in pre‐ and post‐synaptic regions, together with axonal morphology. The MF enrichment highlights a deregulation in gated channels activity, ion channel binding, and neuropeptide binding, along with an involvement of non‐gated receptors and non‐synapses‐related processes. When looking at GO BP, the same pattern is maintained: There is a deregulation in synaptic biology, cell junction assembly, and synapse development, but there is also a presence for non‐neuronal related functions such as cardiac, renal, and neuromuscular development. The same is true for KEGG 2021 enrichment analysis.

When analyzing the 453 terms selectively affected in SH‐SY5Y‐SNCA cells (Figure 4c), the most deregulated cellular components resulted to be the synapses and neuron projections. The main significantly enriched MF term relates to inorganic cation transmembrane transporter activity. Furthermore, a BP enrichment analysis shows a deregulation in nervous system development and neurogenesis. Moreover, KEGG 2021 analysis shows an implication of variable pathways such as cardiac‐ and renal‐related ones but the dopaminergic synapse is the tenth most dysregulated pathway.

Together, these results highlight how SNCA‐AS1 could be of crucial importance in regulating synapses biology through a molecular signature shared with SNCA, and thus, α‐syn's involvement in synaptic processes could possibly be due partially to its modulation by SNCA‐AS1.

### 2.8 Implication for SNCA‐AS1 and SNCA in PD

**FIGURE 5:**
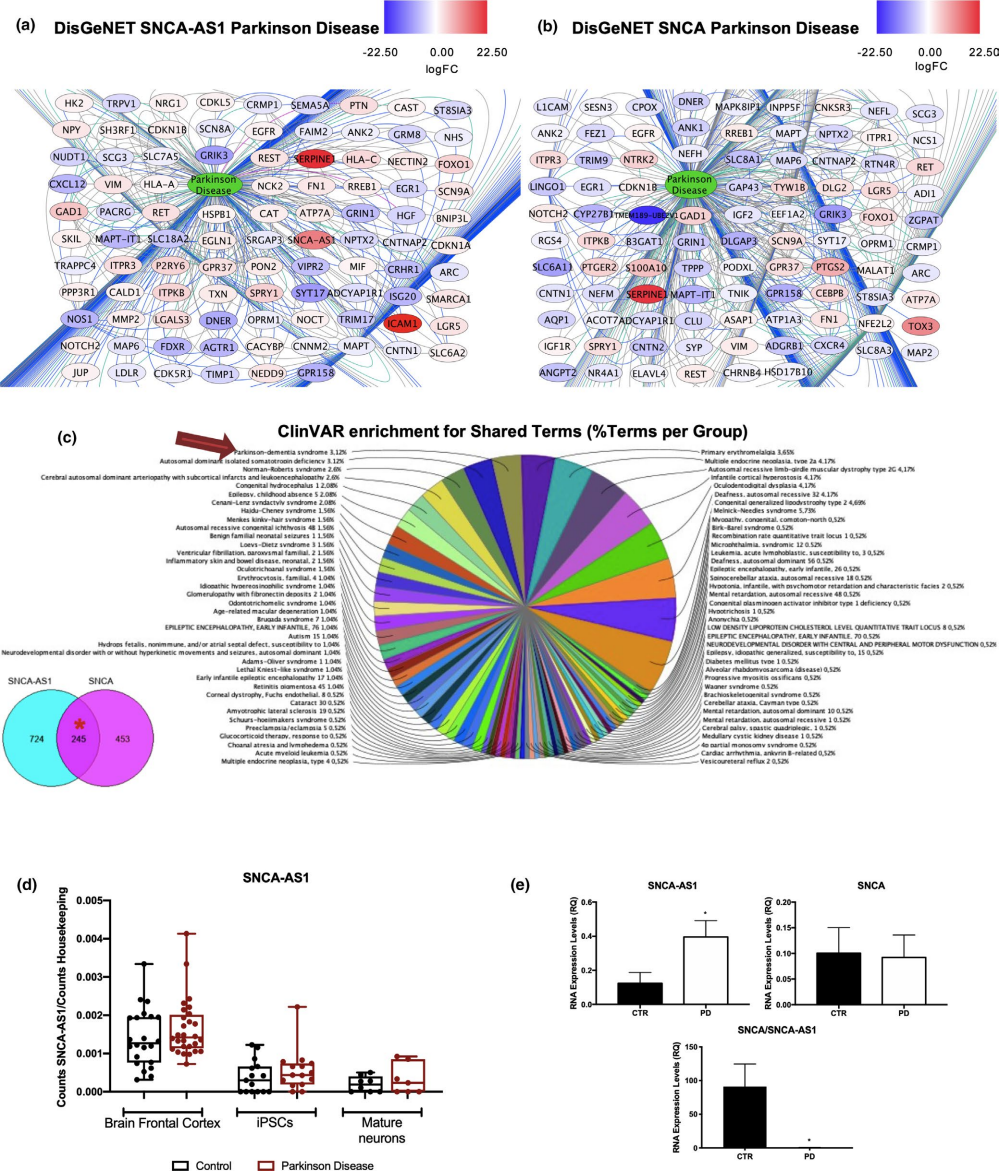
Implications for SNCA‐AS1 and SNCA in Parkinson's disease (PD). DisGeNET analysis shows the terms implicated in Parkinson's disease in SH‐SY5Y‐SNCA‐AS1 (a) or SH‐SY5Y‐SNCA (b) vs SH‐SY5Y, respectively. The lines connecting the genes to the disease term represent the evidences reported in the literature for the terms' implication in the disease. The color scale for the genes represents the genes fold change. (c) ClueGO ClinVAR enrichment in genes deregulated shared between SH‐SY5Y‐SNCA‐AS1 vs SH‐SY5Y and SH‐SY5Y‐SNCA vs SH‐SY5Y. Each pie segment refers to the % of terms present per group. (d) Analysis of SNCA‐AS1 expression in PD datasets present in the literature. (e) Analysis of SNCA‐AS1, SNCA and their ratio in PBMCs of PD patients vs healthy controls (CTR). The analysis was performed by real‐time PCR, and GAPDH was used as housekeeping gene. Data are expressed as mean of the results obtained in 16 patients ±SEM (n = 16; *p < 0.05 vs. CTR)

With the aim to correlate this gene expression evidence with Lewy bodies' pathology, we focused our attention on the identification of sense and antisense regulation of PD‐associated genes. To this end, the DisGeNET tool on Cytoscape was used, allowing the identification of all terms associated with PD. For SH‐SY5Y‐SNCA‐AS1, 95 genes were correlated with PD (Figure 5a), and their specific annotation on peer‐reviewed literature evidence is reported in Table S12. The most described term is MAPT, which codes for the TAU protein. When considering SH‐SY5Y‐SNCA, 95 genes were correlated with PD (Figure 5b), and their specific annotation on peer‐reviewed literature evidence is reported in Table S13, and MAPT is also present in this case as the most PD‐characterized gene.

With the aim to identify whether it was SNCA‐AS1‐specific genes, shared genes, or SNCA‐specific genes which were more likely to lead to the development of PD pathogenesis, the three classes of terms were analyzed with the ClinVAR database. This database allows for the identification of all possible diseases associated with a certain gene set. Remarkably, the most significant correlation with PD was observed in the shared terms dataset, where out of all the possible disease associated a correlation was obtained with “Parkinson‐dementia Syndrome” (3,12% terms per group) (Figure 5c). This is interesting to report as there could be a deregulation in the overlapping SNCA‐AS1 and SNCA regulated pathways in PD, with a specific focus on the processes which are affected by their synergic regulation. To validate the implication for SNCA‐AS1 in PD, we assessed its expression in PD‐related datasets (Dumitriu et al., 2016; Nido et al., 2020; Schulze et al., 2018) and we found that in all analyzed conditions (PD brain frontal cortex, PD iPSCs, and PD mature neurons) SNCA‐AS1 was upregulated (Figure 5d). To provide initial evidence for this claim in PD patients, the expression levels of SNCA‐AS1 and SNCA were analyzed in PBMCs of PD‐affected patients vs. control (n = 16) (Figure 5e). Remarkably, SNCA‐AS1 results upregulated, SNCA's mRNA level does not change but there is a significant reduction in the sense/antisense ratio present in PD‐affected patients (Figure 5d). Lastly, when searching for genetic databases in the AnnoLnc2 database which annotates human single nucleotide polymorphisms (SNP) along with their position and the associated traits, 45 out of 48 found SNPs emerge as associated with PD (Table S14).

### 2.9 SNCA‐AS1 downregulation leads to a concordant downregulation of SNCA and an impact on synapses‐related genes

**FIGURE 6:**
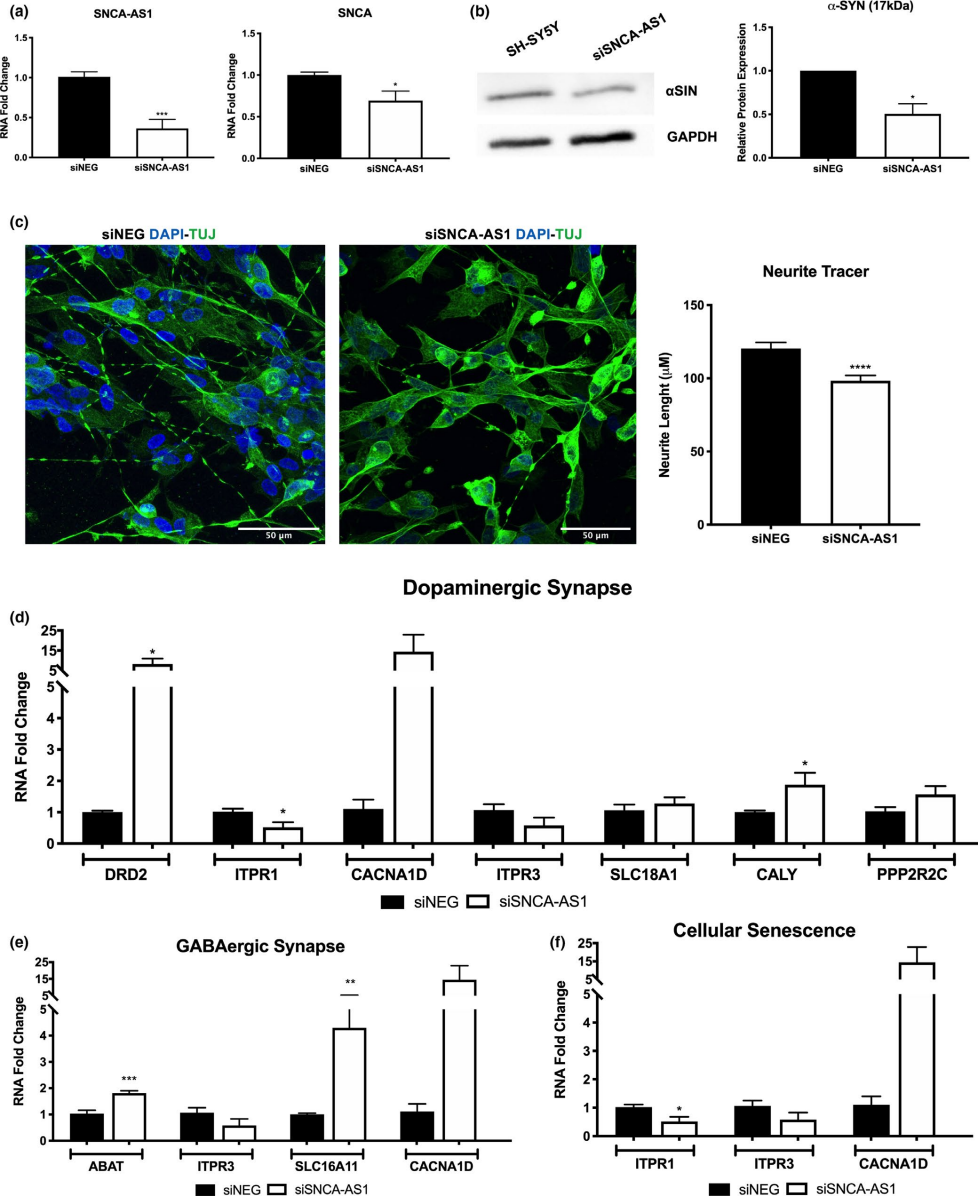
SNCA‐AS1 downregulation reduces α‐syn expression and synaptic length in differentiated SH‐SY5Y cells. (a) Real‐time PCR expression of SNCA‐AS1 and SNCA when SNCA‐AS1 expression was knocked‐down with RNA interference technique (siSNCA‐AS1) in SH‐SY5Y cells differentiated for 7 days with retinoic acid. Results are an average of 4 independent experiments, each one performed in duplicates (N = 8, *p < 0.05, ***p < 0.001 vs siNEG). (b) Western blot analysis of α‐syn expression when SNCA‐AS1 expression was knocked‐down (siSNCA‐AS1). Results are an average of 3 independent experiments (N = 3, *p < 0.05 vs siNEG). The graph reports the quantification of the WB bands. (c) Representative immunofluorescence images of TUJ expression in SH‐SY5Y differentiated for 7 days with retinoic acid in presence of siNEG and siSNCA‐AS1. Nuclei were labeled in blue (DAPI). Scale bar 50 μm. Neurite Tracer analysis is presented as mean of the quantification of 10 cells performed in 3 different fields for each condition, in 3 independent experiments ±SEM (n = 90, ***p < 0.001 vs. siNEG). Real‐time PCR analysis of genes correlated with Dopaminergic Synapse (d), GABAergic Synapse (e), and Cellular Senescence (f) in siSNCA‐AS1 vs siNEG. Results are an average of 4 independent experiments performed in duplicates (N = 8, *p < 0.05, **p < 0.01, ***p < 0.001 vs. siNEG)

Our results have so far shown that the overexpression of SNCA‐AS1 upregulates SNCA mRNA and protein. Moreover, our results reported a reduced expression of markers associated with synaptic plasticity. To validate whether these results were specifically due to SNCA‐AS1 overexpression, we inhibited SNCA‐AS1 in SH‐SY5Y differentiated for 7 days with retinoic acid (Figure 6). SNCA‐AS1 was efficiently knocked‐down and SNCA expression was concordantly downregulated (Figure 6a). α‐syn expression was also significantly downregulated after SNCA‐AS1 inhibition (Figure 6b). We also validated the impact of SNCA‐AS1 downregulation on neurite length and synaptogenesis, and we found a reduced neurites extension (Figure 6c). Lastly, we found that the expression trends of genes implicated in dopaminergic synapses (Figure 6d), GABAergic synapses (Figure 6e), and cellular senescence (Figure 6f) were opposite to those observed when SNCA‐AS1 was overexpressed (Figure 3f‐h).

## 3 DISCUSSION

More and more research is now establishing how PD is an evolution of the aging process, with numerous overlapping mechanisms (Bobela et al., 2015; Rodriguez et al., 2015). Indeed, α‐syn, crucial for PD pathogenesis, can promote the accumulation of pathogenic proteins and lead to an impairment of the ubiquitin‐proteasome system, ultimately resulting in dopaminergic degeneration in both PD and the aging brain (Rodriguez et al., 2015). Knowledge on α‐syn's physiological mechanisms of action still requires clarifications, and modulation of its coding gene SNCA has been shown to be implicated in aging and disease pathogenesis. Recently, research efforts are focusing on lncRNAs, known for their modulatory role at different levels of gene expression and for their implications in both aging and PD‐ associated neurodegenerative processes (Elkouris et al., 2019; Lyu et al., 2019; Pereira Fernandes et al., 2018). The aim of this work was thus to focus on investigating the potential disruption in gene expression obtained when SNCA or its natural antisense transcript, SNCA‐AS1, are overexpressed in SH‐SY5Y. We demonstrated that SNCA‐AS1 overexpression leads to the increase in SNCA's expression, in both its transcript and protein product. Furthermore, the downregulation of SNCA‐AS1 leads to an opposite profile, confirming its importance in regulating SNCA and, consequently, α‐syn expression. RNA‐Seq analysis allowed us to identify a significant number of DE RNAs in both cell lines, indicating a significant impact of these two transcripts on gene expression.

Through KEGG enrichment analysis we obtained the detailed profile of the pathways in which these genes were implicated. Particularly, GSEA analysis showed a reduced expression of terms associated with neurodegenerative diseases in SH‐SY5Y‐SNCA and, together with g:Profiler and EnrichR, allowed the identification of a strong enrichment for SNCA‐AS1 and SNCA in pathways concerning synapses and aging as terms involved in this process were amongst the top enriched. GO enrichment analysis further implicated SNCA in synapses and SNCA‐AS1 to a lesser extent, as in this case there is again also a strong involvement of cellular interaction with the surrounding environment. In both SH‐SY5Y‐SNCA‐AS1 and SH‐SY5Y‐SNCA, there is a reduction in genes associated with synaptic plasticity, such as c‐fos, implicated in the aging process and long‐term synaptic plasticity (Jaworski et al., 2018). Moreover, in both contexts, there is a decrease in the expression of the gene that codes for VMAT. Interestingly, multiple studies report how this transporter is dysfunctional in PD and implicated in aging (Meyza et al., 2007). A significant disruption of GABAergic synapse was observed both in SH‐SY5Y‐SNCA‐AS1 and in SH‐SY5Y‐SNCA, and the downregulations in these specific genes have been reported also in the aging brain (Rozycka & Liguz‐Lecznar, 2017). The cellular senescence pathway was also found deregulated, and SNCA‐AS1 could thus be implicated in aging‐related processes. As we demonstrated that SNCA's expression is increased when SNCA‐AS1 is overexpressed, it was not surprising to identify several shared terms between the two deregulated gene sets. A global analysis allowed us to perform a primary characterization of the potential genes' involvement, but it did not discriminate between those pathways, if any, selectively due to SNCA‐AS1 overexpression, to SNCA mRNA overexpression, or to a mechanism of action shared between the two and due to the overlapping deregulated genes. When discerning the two datasets, we found that SNCA‐AS1 involvement in synapses biology was predominant when analyzing the terms shared among the two datasets, while “SNCA‐only” genes also independently affect synapses biology. This highlights that SNCA‐AS1 could be a player in synaptic modulation through its modulation of SNCA and α‐syn. It is thus also possible to speculate that α‐syn's involvement in synaptic modulation could be partially due to a common pathway initiated or shared with SNCA‐AS1. The presence of PD‐related genes in both datasets also shows that SNCA‐AS1 could be a direct player in the pathogenesis of the diseases, and the evidence reported in PBMCs of PD patients shows that the SNCA/SNCA‐AS1 ratio could be what is disrupted in the disease and could prove to be a relevant biomarker for the pathology.

Even if further research is needed, the findings here reported demonstrate a strong impact for SNCA‐AS1 in the aging process and ultimately in PD, suggesting that this lncRNA may be even more crucial than we thought.

## 4 EXPERIMENTAL PROCEDURES

### 4.1 Cell culture and expression modulation

Human neuroblastoma SH‐SY5Y cells (ATCC) were grown in DMEM low glucose (Gibco) supplemented with 15% of fetal bovine serum (Euroclone), 1% L‐glutamine (Gibco), and 1% penicillin/streptomycin (Gibco) at 37°C in a 5% CO_2_ atmosphere. For SNCA‐AS1 stable transfection, a pCMV6‐AC‐RFP construct containing gentamicin‐disulfate G418 resistance was used (OriGene). For SNCA stable transfection, a custom pCMV6‐AC‐GFP construct containing G418 resistance was used (alpha Synuclein SNCA NM_000345 Human Tagged ORF Clone, RG210606, OriGene). The plasmids were transformed in *E. Coli*, where individual colonies were checked for successful ligations by sequencing. SH‐SY5Y cells were subsequently stably transfected with FugeneHD transfection reagent (Roche) and kept in OPTIMEM (Gibco). After 48 h incubation, the culture medium was changed, and cells were cultured with selective medium containing G418 (Gibco). The cell lines were successfully obtained as can be observed by GFP and RFP expression (EVOS FL, Cell Imaging System AMG; Thermo Fisher) and real‐time PCR analysis (Figure S8).

For silencing studies, 2 days before transfection, SH‐SY5Y cells were plated in standard growth medium. Transfection was performed with Lipofectamine^®^ RNAiMAX Reagent (Invitrogen) and siRNA agent (Invitrogen) diluted in the appropriate volume of OPTIMEM (Gibco) and following standard protocol, for 72 h. Samples were then collected for further analyses.

### 4.2 RNA secondary structure prediction

RNA secondary structure was predicted using the Geneious software (Geneious version 2020.1 created by Biomatters. Available from https://www.geneious.com) based on Vienna RNA Fold (Lorenz et al., 2011) with default settings.

### 4.3 Transcription factors' prediction

Transcription factors binding sites were predicted through Ciiider software. Ciiider analysis was performed using the human GRCh38 genome and the 2020 JASPAR core non‐redundant vertebrate matrices (Fornes et al., 2020). All promoter regions were defined as spanning −1500 bases to +500 bases relative to the transcription start site.

### 4.4 Phylogenetic analysis

Phylogenetic analysis was performed using Geneious software (Geneious version 2020.1 created by Biomatters. Available from https://www.geneious.com). The SNCA‐AS1 sequence was used as query to search the sequences with high similarity in databases using Megablast (Chen et al., 2015). Sequences with high pairwise identity were chosen and used as input for multiple alignment. Multiple alignment was performed using Clustal Omega (Sievers & Higgins, 2014). The final sequence alignment was used to perform phylogenetic analysis employing the distance‐based NJ method and the ML implemented in the PHYML program (Guindon et al., 2010). The genetic distance for NJ method was calculated through the Tamura‐Nei model.

### 4.5 RNA extraction

Total RNA from SH‐SY5Y, SH‐SY5Y‐SNCA‐AS1, and SH‐SY5Y‐SNCA was isolated using TRIzol Reagent (Invitrogen) following standard protocol. RNA quality was assessed using a spectrophotometer (NANOPhotometer^®^ NP80; IMPLEN) and a 2100 Bioanalyzer (Agilent RNA 6000 Nano Kit).

Cytoplasmic and nuclear RNA fractions were extracted using the Cytoplasmic & Nuclear RNA Purification Kit (Norgen Biotek Corp) according to the manufacturer's instructions.

### 4.6 Real‐Time PCR

Total RNA (1 μg) was reverse transcribed using iScript cDNA synthesis kit (Bio‐Rad) according to the manufacturer's instructions. Using gene sequences available from NCBI for target genes (http://www.ncbi.nlm.nih.gov/nucleotide), PCR oligonucleotide primers were selected and are reported in Table S15. This was done with the NCBI's Primer‐BLAST tool. Real‐time PCR was performed with StepOnePlus^TM^ Real‐Time PCR System (Invitrogen) using SSOSYBR Green Supermix (Bio‐Rad). Genes were quantified in triplicates; GAPDH was used as housekeeping gene. Gene expression was calculated using the 2^−ΔΔ^ *^C^* ^*t*^ method.

### 4.7 Droplet digital PCR

Total RNA (200 ng) was reverse transcribed using iScript cDNA synthesis kit (Bio‐Rad) according to the manufacturer's instructions. Droplet digital PCR (ddPCR) reaction mixture contained 2× EVAGreen Supermix (Bio‐Rad), 100 nM of each forward and reverse primers (sequence reported in Table S15) and 10 ng of cDNA. Droplet emulsion was generated using QX 200 droplet generator (Bio‐Rad) and analyzed with QX 200 droplet reader (Bio‐Rad).

### 4.8 Library preparation for RNA‐seq and bioinformatic data analysis

500 ng of total RNA from SH‐SY5Y, SH‐SY5Y‐SNCA‐AS1, and SH‐SY5Y‐SNCA was used to prepare libraries with the SENSE Total RNA‐Seq Library Prep Kit (Lexogen) and sequenced by Illumina NextSeq 500 sequencer. Qualities of sequencing libraries were assessed by 2100 Bioanalyzer with DNA1000 assay (Agilent) and quantified with Qubit dsDNA HS Assay Kit (Invitrogen). FastQ files were generated via Illumina bcl2fastq2 starting from raw sequencing reads produced by Illumina NextSeq 500 sequencer (Version 2.17.1.14‐ http://support.illumina.com/downloads/bcl‐2fastq‐conversion‐software‐v217.html). The raw data obtained from the RNA‐Seq analysis are deposited on the Gene Expression Omnibus repository: GSE183410 for SH‐SY5Y and GSE186255 for SH‐SY5Y‐SNCA and SH‐SY5Y‐SNCA‐AS1. Gene and transcript intensities were computed using STAR/RSEM software (Li & Dewey, 2011) using Gencode Release 27 (GRCh38) as a reference, using the “stranded” option. Differential expression analysis was performed using R package DESeq.2 (Love et al., 2014). Coding and non‐coding genes were considered differentially expressed and retained for further analysis with |log_2_(SH‐SY5Y‐SNCA‐AS1/SH‐SY5Y)| ≥1 and a FDR ≤0.1 and |log_2_(SH‐SY5Y‐SNCA/ SH‐SY5Y)| ≥1 and a FDR ≤0.1.

### 4.9 Pathway analysis and Gene Ontology

Gene set enrichment analysis was performed on clusterProfiler R package (Yu et al., 2012). Gene set from Molecular Signature databases such as curated gene set (C2) and ontology gene sets (C5) and a *p*‐value cutoff <0.05 were considered for this analysis (Yu et al., 2012). Moreover, functional enrichment analysis was performed on coding DE RNAs using the EnrichR webtool (Kuleshov et al., 2016) and g:Profiler, ranking terms according to their absolute fold change and using a Bonferroni–Hochberg FDR of 0.05 as threshold. The R software was used to generate Volcano plots (Zhu et al., 2019), Dotplot graphs (ggplot2 library), and Pathview graphs (Pathview library [Luo & Brouwer, 2013]). All other representations of functional enrichment were generated using the Cytoscape software (Shannon et al., 2003) and the DisGeNET plugin (Piñero et al., 2017).

### 4.10 Western blot

Cell protein extracts were obtained by means of RIPA lysis buffer. Equal amounts of solubilized proteins were heated in Laemmli sample buffer (Bio‐Rad) containing 2‐βmercaptoethanol (70 mM, Sigma), separated by SDS‐PAGE gel 10% and electroblotted onto a nitrocellulose membrane (GE Healthcare, Amersham™). Membranes were fixed for 30 min in 0.4% paraformaldehyde (Lee & Kamitani, 2011). Membranes were blocked in 5% slim milk (diluted in TBS with 0.05% Tween‐20) and probed with the appropriate primary antibody against α‐syn (AB138501; abcam) and GAPDH (2118; Cell Signaling) overnight at 4°C. The membrane was then incubated with specific secondary antibody Peroxidase AffiniPure Goat Anti‐Rabbit/Mouse IgG (Jackson). Proteins were visualized by means of an enhanced chemiluminescence detection system (ECL™; Amersham). After acquisition by a GelDoc™ image capture system (Kodak), proteins were quantified using ImageJ software.

### 4.11 Immunofluorescence and confocal microscopy

SH‐SY5Y cells were seeded on ethanol‐washed glass coverslips, maintained in the appropriate culture medium, and processed for immunocytochemistry following an already described protocol (Marfia et al., 2011). Briefly, cells were fixed with 4% paraformaldehyde (Life Technologies) for 20 min at room temperature, then washed with PBS and incubated overnight at 4°C in PBS containing 10% normal goat serum (Thermo Fisher), 0.3% Triton X‐100 (BDH) with the anti‐TUJ antibody (GTX631836; Genetex). After thorough washing with PBS and 10% NGS, cells were reacted for 45 min (room temperature) with the appropriate secondary antibody (Alexa Fluor^®^ 488 and 546, Life Technologies). Nuclei were stained with DAPI (1 µg/ml final concentration, 10 min at room temperature) mounted using the FluorSave Reagent (Calbiochem; Merck Chemical), and analyzed by confocal microscopy (Confocal laser scanning microscopy platform Leica TCS SP8; Leica Microsystems). In control experiments, primary antibodies were omitted and replaced with equivalent concentrations of unrelated IgG of the same subclass.

### 4.12 Neurite length analysis

For immunofluorescence images, TUJ was used to mark the cytoskeleton and neurite extension, and the Simple Neurite Tracer plugin of Fiji software was used (Longair et al., 2011).

### 4.13 Study subjects

Sixteen PD patients and sixteen age‐ and sex‐matched healthy controls were recruited after obtaining written informed consent. PD patients underwent clinical and neurologic examination at IRCCS National Neurological Institute “C. Mondino” (Pavia, Italy). All patients were diagnosed with PD as defined by Movement Disorder Society clinical diagnostic criteria (Postuma et al., 2015). The control subjects were recruited at the Transfusional Service and Centre of Transplantation Immunology, Foundation San Matteo, IRCCS (Pavia, Italy). The study protocol was approved by the Ethical Committee of the National Neurological Institute “C. Mondino”, IRCCS (Pavia, Italy). Before being enrolled, the subjects participating in the study signed an informed consent form (Protocol n° p‐20170001758).

### 4.14 Isolation of human PBMCs

Peripheral blood mononuclear cells were isolated by centrifugation on a Ficoll‐Histopaque layer (Sigma‐Aldrich), and cells were used for subsequent RNA extraction.

### 4.15 Statistical analysis

Data are expressed as mean ± SEM. The statistical analysis was performed with Student's t test when two datasets were considered, and one‐way ANOVA with Bonferroni's post‐test when three datasets were considered. The Prism 7 software (GraphPad Software Inc.) was used assuming a *p*‐value <than 0.05 as the limit of significance.

## CONFLICT OF INTEREST

The authors declare that they have no conflict of interest.

## AUTHOR CONTRIBUTIONS

FR involved in design, conception, data generation, data acquisition, data interpretation, and manuscript writing. CP involved in design, data generation, data acquisition, data interpretation, and data analysis. LM involved in bioinformatic data analysis and interpretation. RL involved in data generation and data acquisition. BB involved in data generation, data acquisition, and discussion. RZ involved in patient selection and clinical data generation. MTR involved in supervision of the work and manuscript revision. SG involved in transcriptomic data interpretation. CC involved in supervision of the work, draft and manuscript revision and financial support. GVZ involved in supervision of the work, manuscript revision, and financial support. SC involved in conception and design, supervision of the work, data interpretation, manuscript writing and revision.

## Supporting information

Figure S1

Figure S2

Figure S3

Figure S4

Figure S5

Figure S6

Figure S7

Figure S8

Table S1

Table S2

Table S3

Table S4

Table S5

Table S6

Table S7

Table S8

Table S9

Table S10

Table S11

Table S12

Table S13

Table S14

Table S15

## Data Availability

The raw data obtained from the RNA‐Seq analysis are deposited on the Gene Expression Omnibus repository: GSE183410 for SH‐SY5Y and GSE186255 for SH‐SY5Y‐SNCA and SH‐SY5Y‐SNCA‐AS1.
