# Supplementary figures and images for: α‐Synuclein antisense transcript SNCA‐AS1 regulates synapses‐ and aging‐related genes suggesting its implication in Parkinson's disease
Source: Aging Cell. 2021 Nov 19;20(12):e13504. doi: 10.1111/acel.13504 (PMC8672788; doi:10.1111/acel.13504)

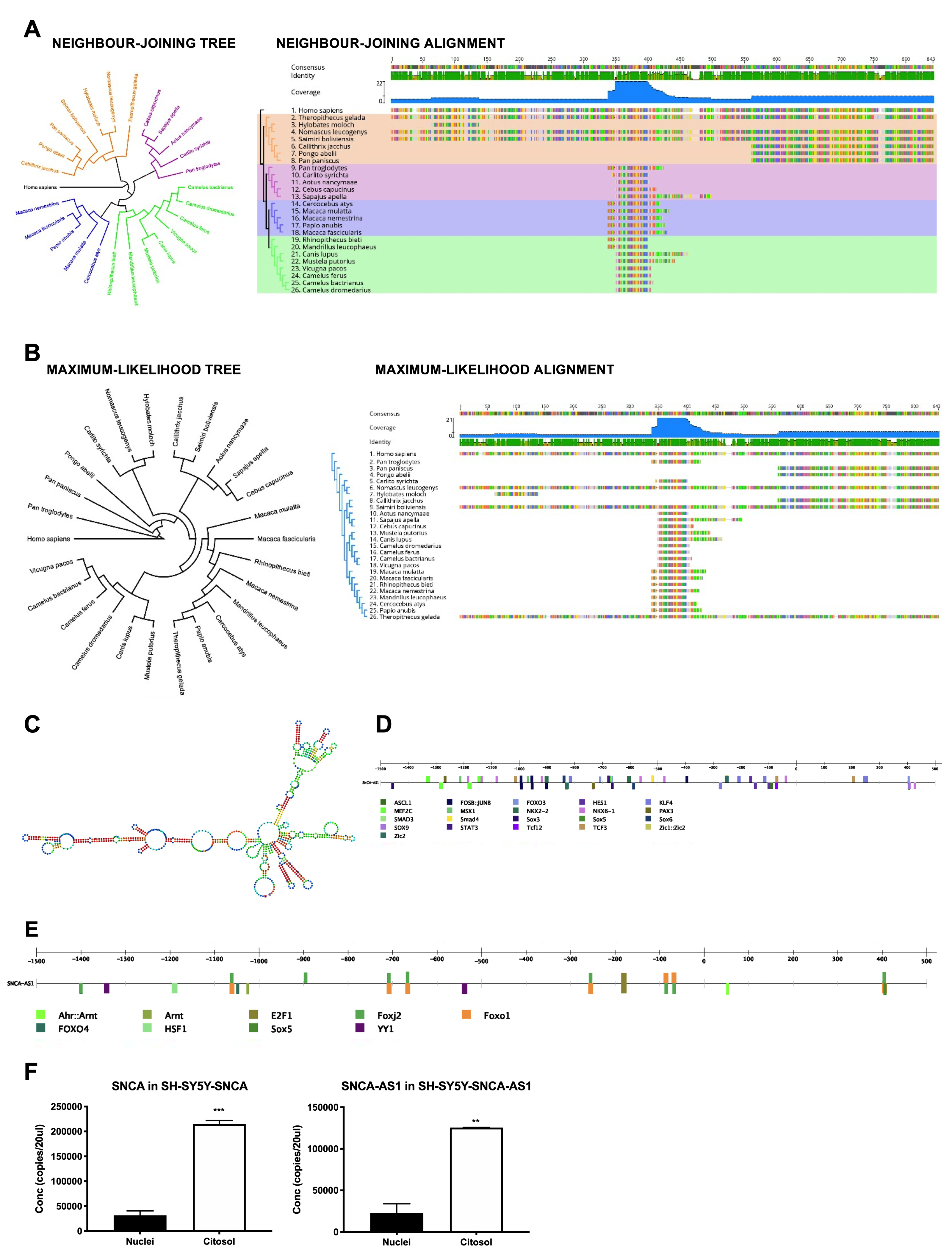

Supplement: Supplementary file 1 — Figure S1 [file ACEL-20-e13504-s001.tiff]

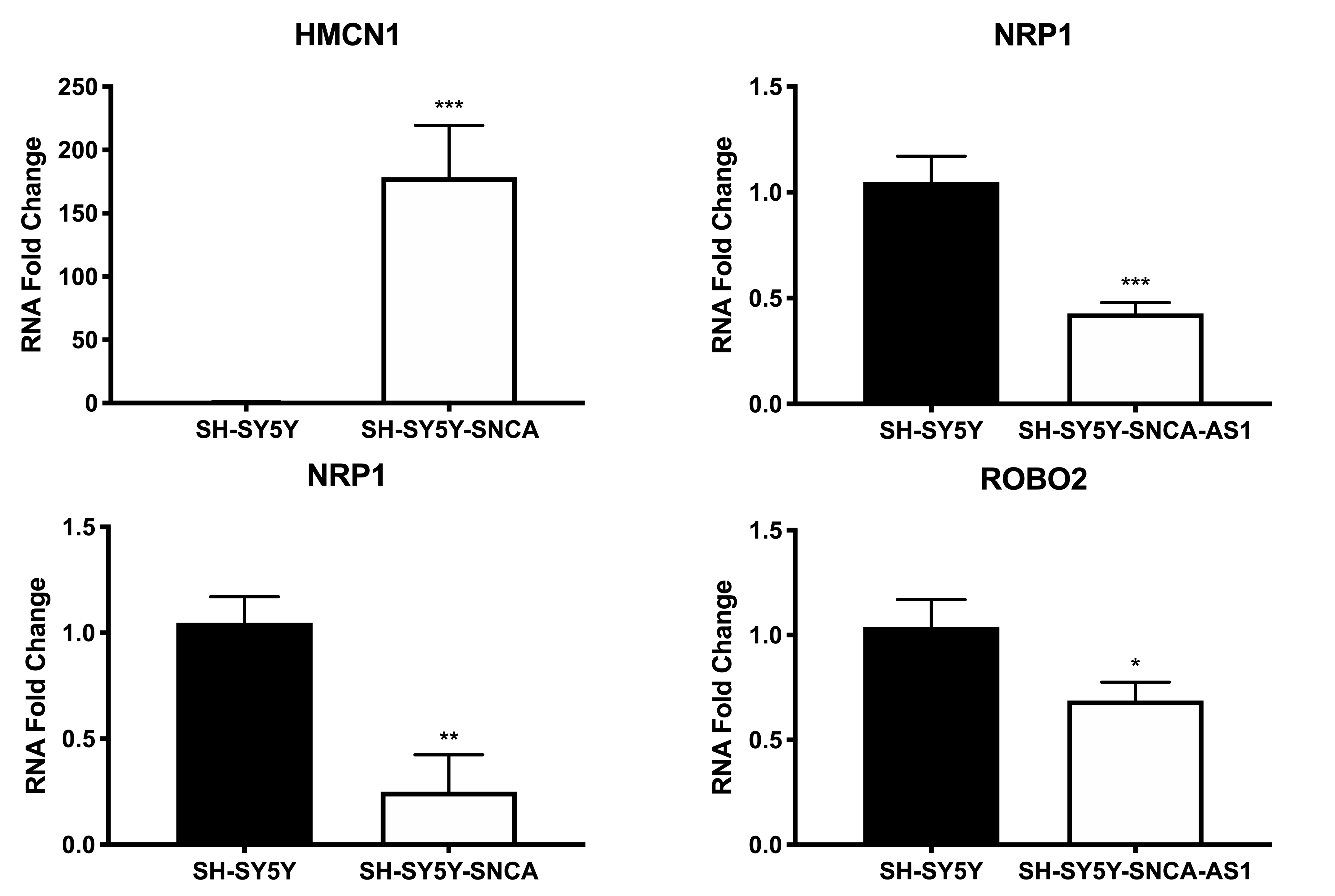

Supplement: Supplementary file 2 — Figure S2 [file ACEL-20-e13504-s014.tiff]

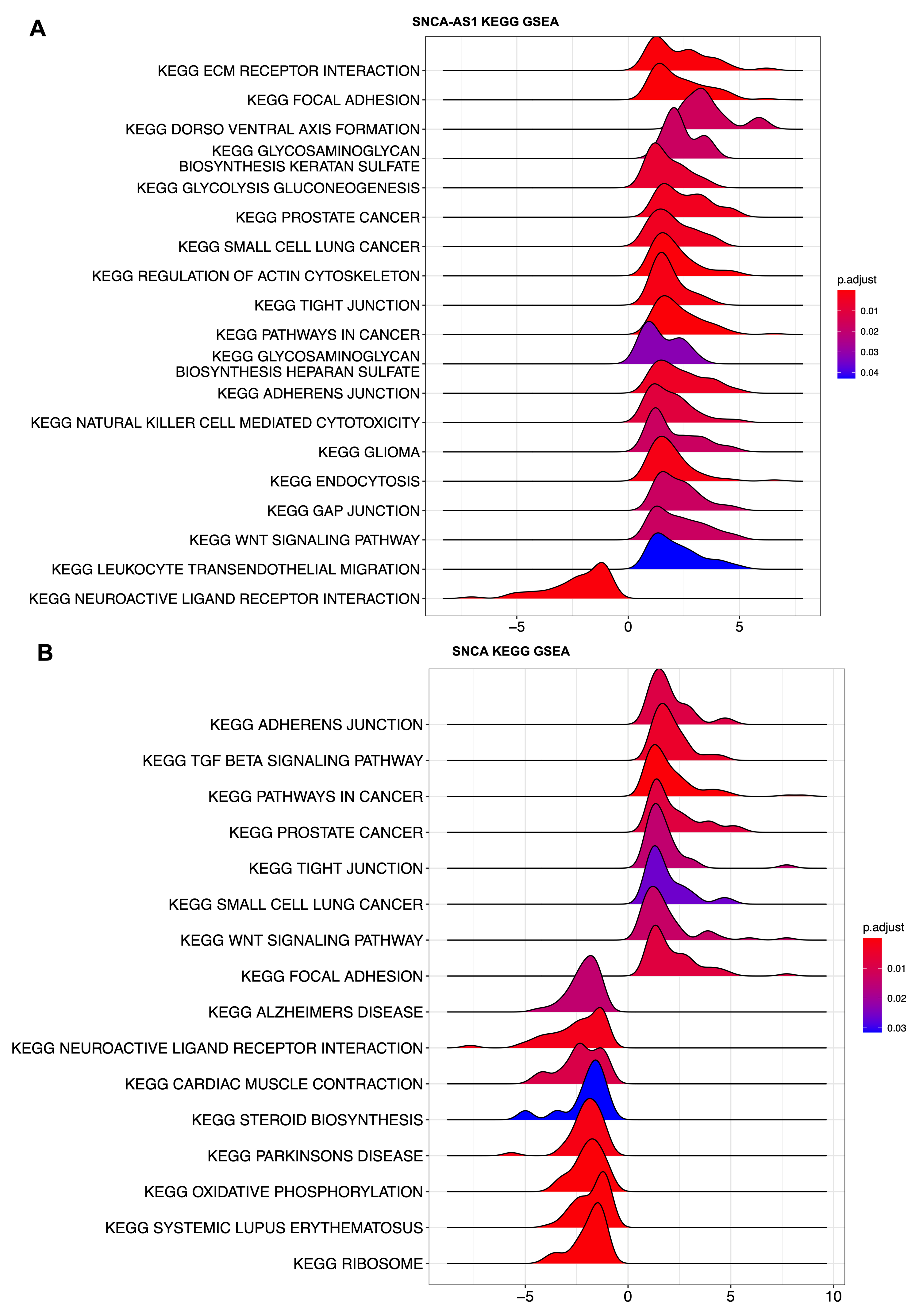

Supplement: Supplementary file 3 — Figure S3 [file ACEL-20-e13504-s016.tiff]

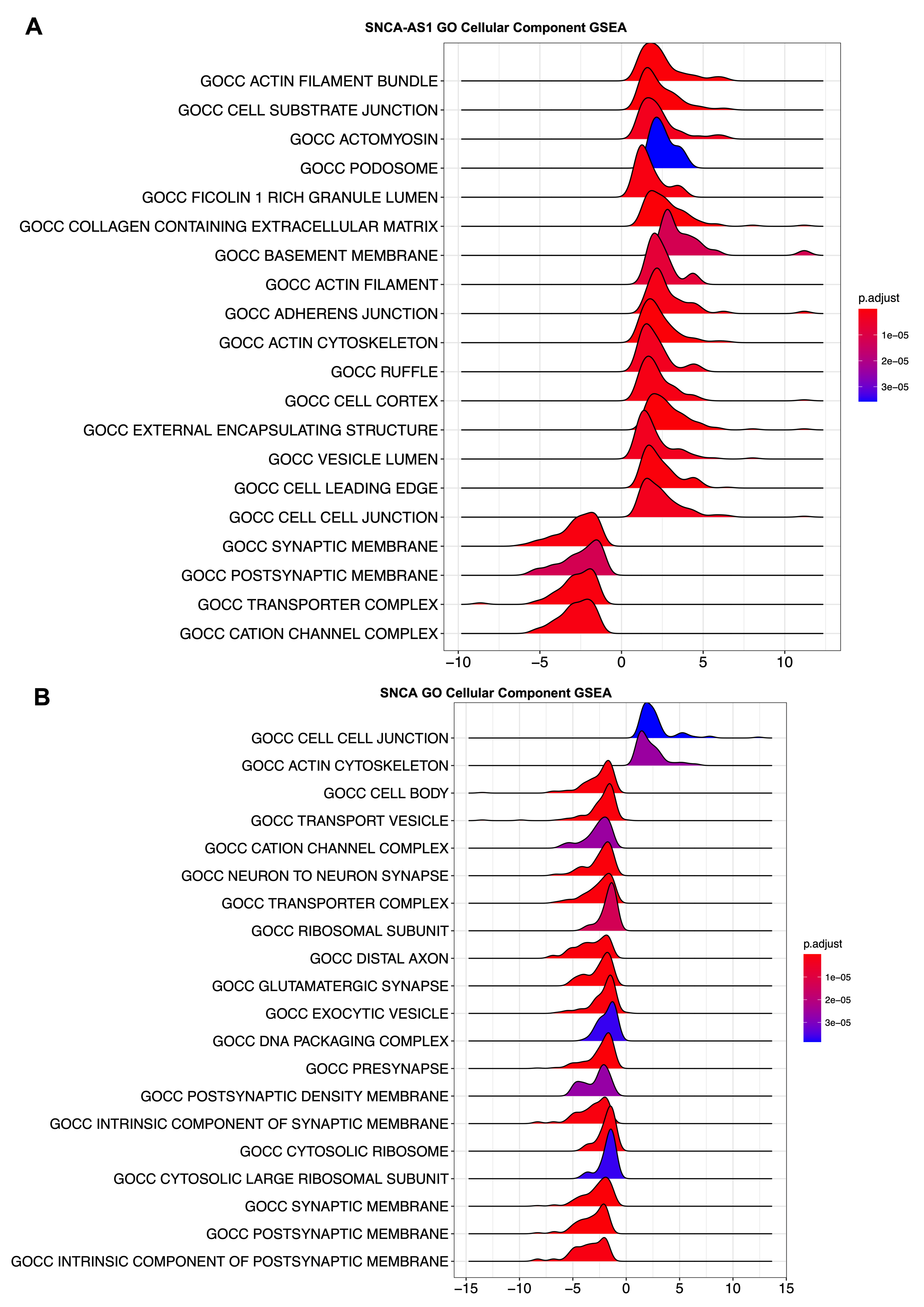

Supplement: Supplementary file 4 — Figure S4 [file ACEL-20-e13504-s004.tiff]

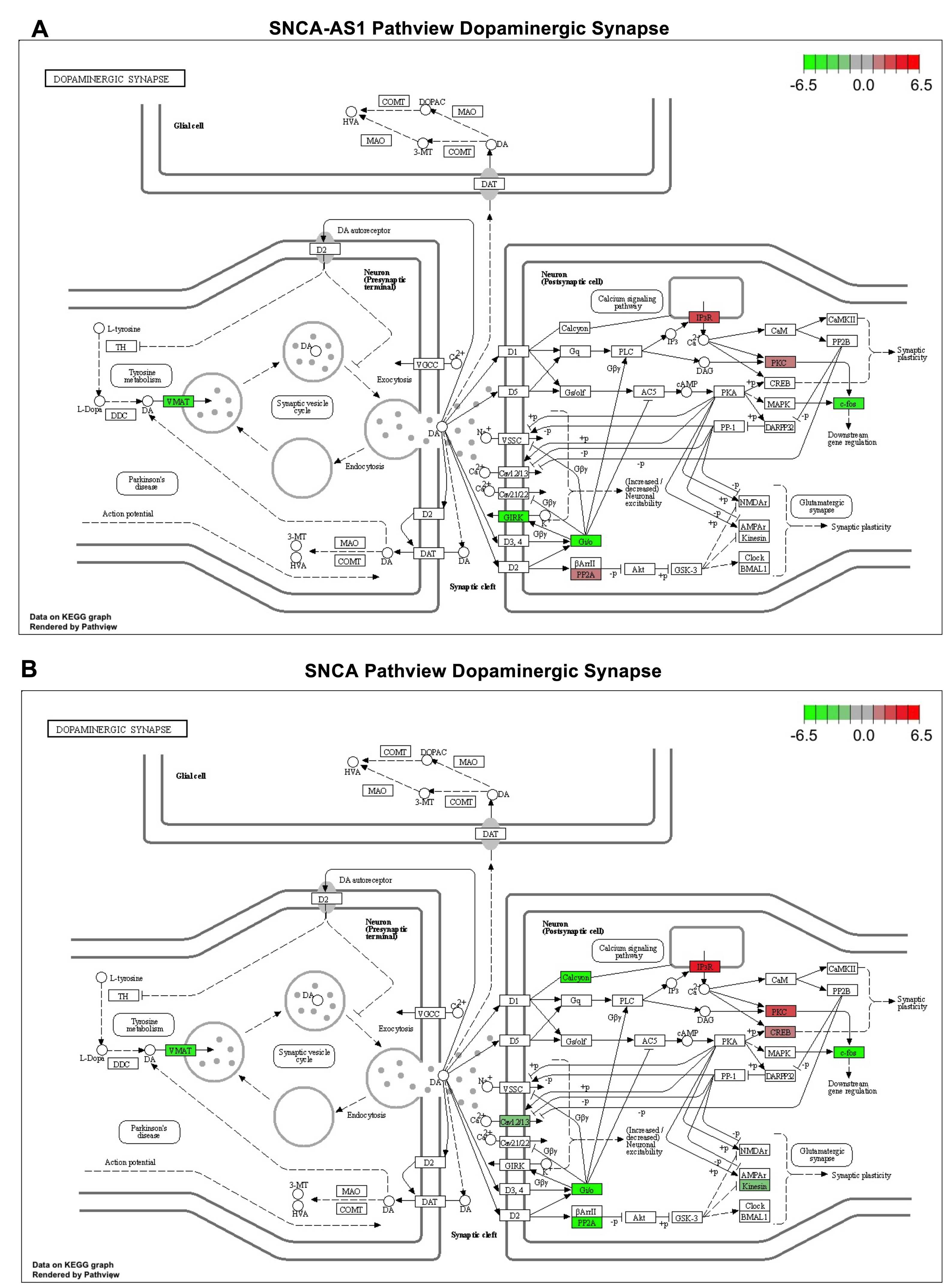

Supplement: Supplementary file 5 — Figure S5 [file ACEL-20-e13504-s003.tiff]

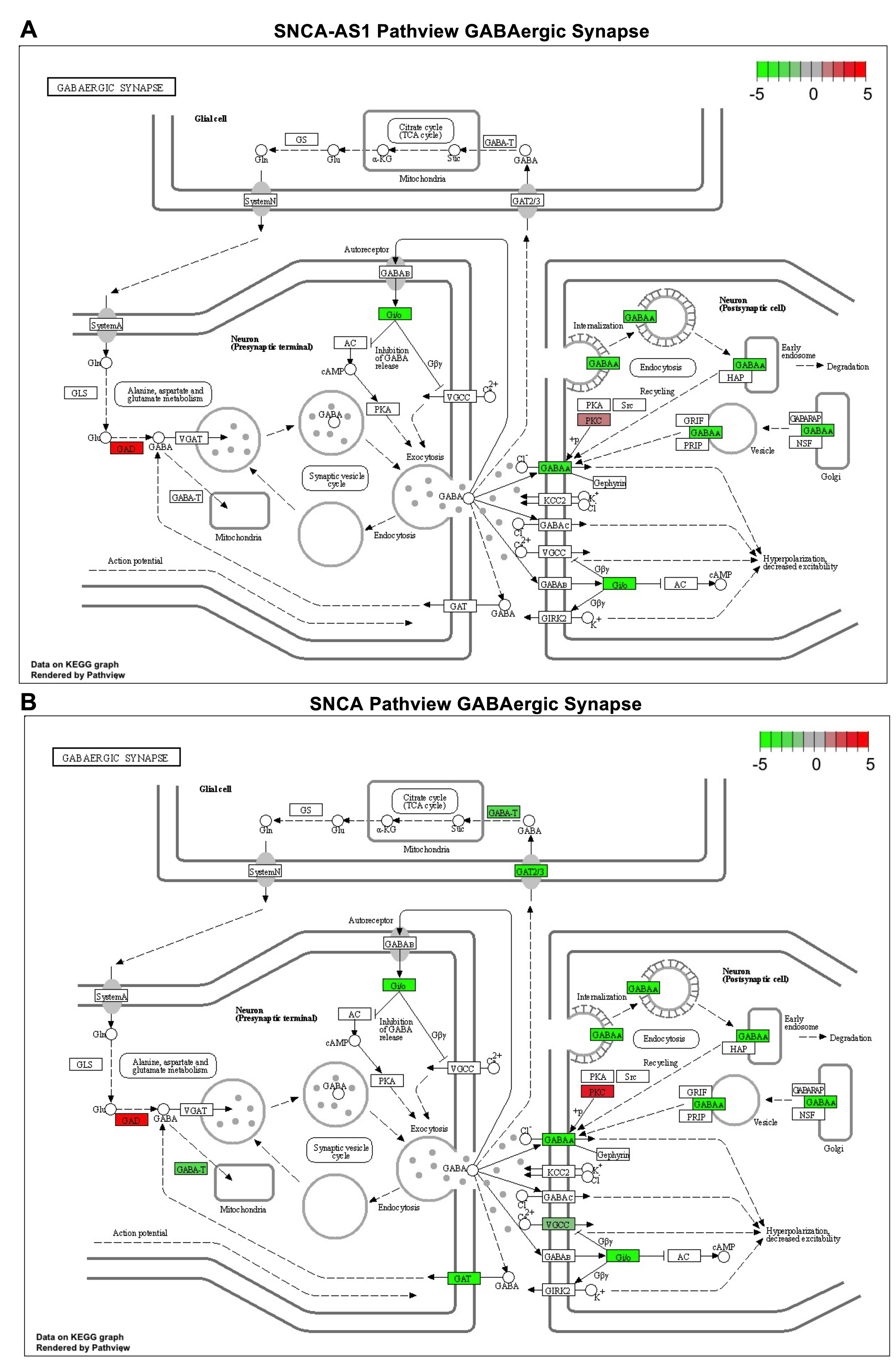

Supplement: Supplementary file 6 — Figure S6 [file ACEL-20-e13504-s012.tiff]

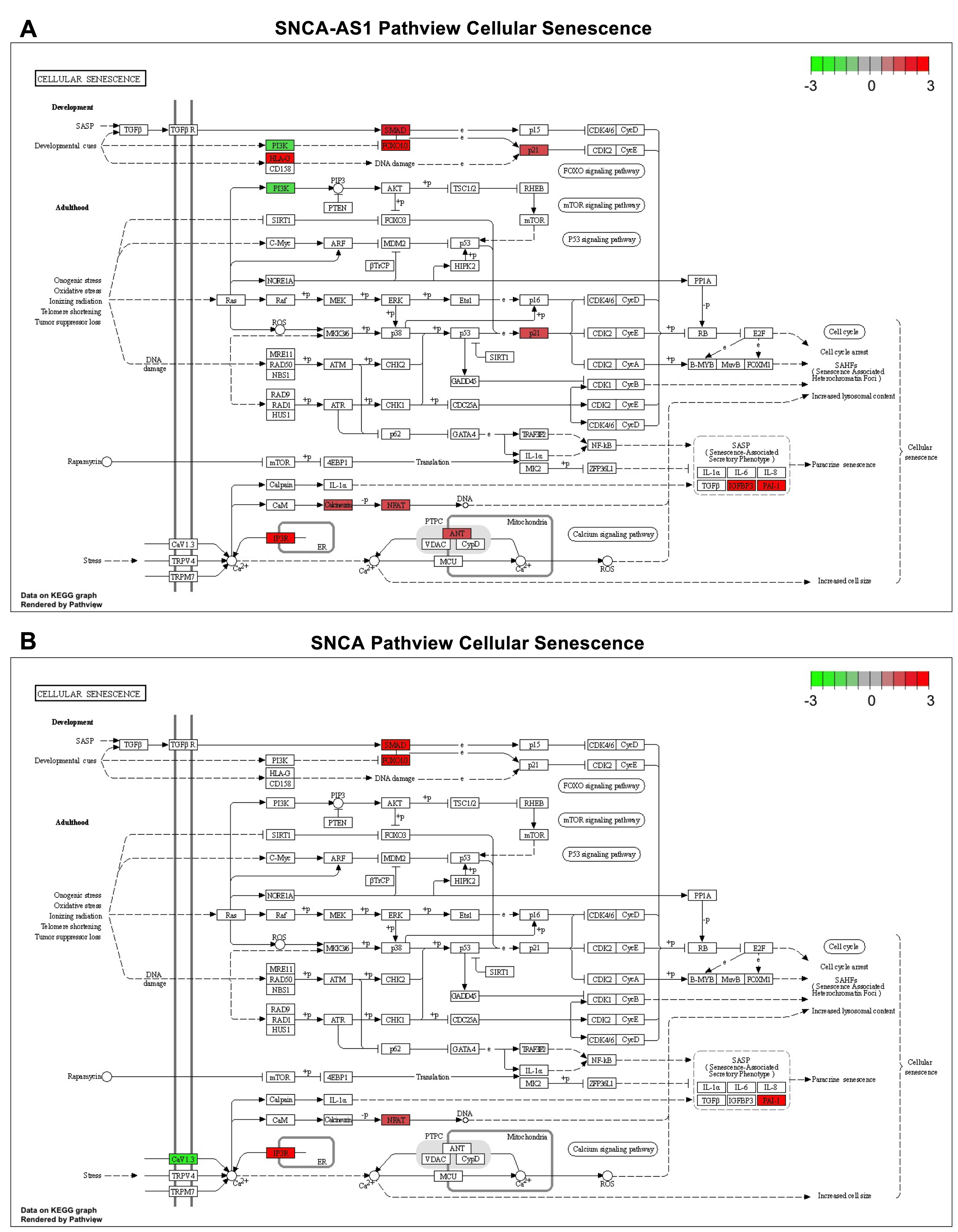

Supplement: Supplementary file 7 — Figure S7 [file ACEL-20-e13504-s018.tiff]

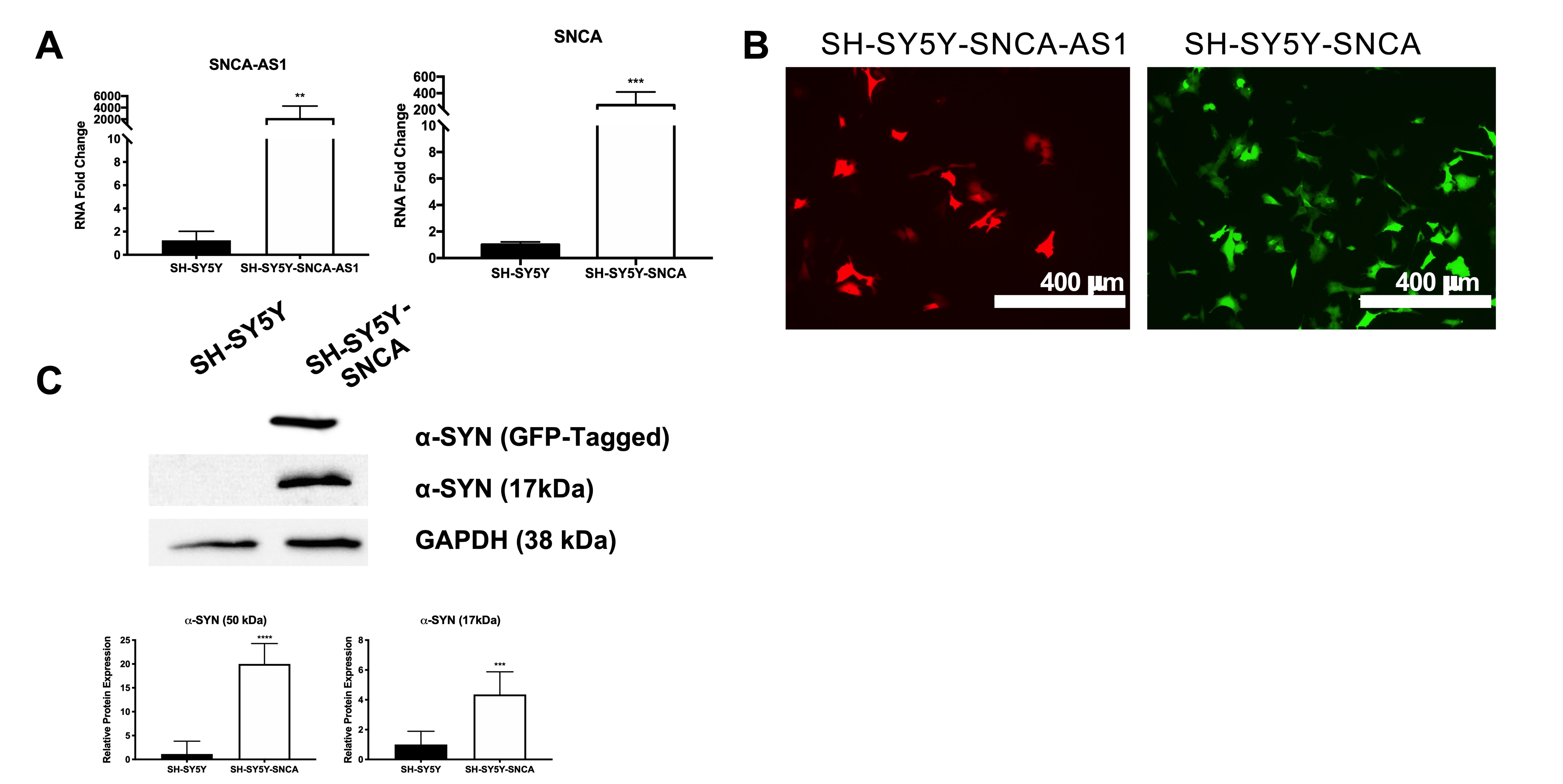

Supplement: Supplementary file 8 — Figure S8 [file ACEL-20-e13504-s007.tiff]
