# Supplementary material for: α‐Synuclein antisense transcript SNCA‐AS1 regulates synapses‐ and aging‐related genes suggesting its implication in Parkinson's disease
Source: Aging Cell. 2021 Nov 19;20(12):e13504. doi: 10.1111/acel.13504 (PMC8672788; doi:10.1111/acel.13504)
Supplement: Supplementary file 23 — Table S15 [file ACEL-20-e13504-s011.docx]

**Table S15.** List of Primer sequences for Real Time PCR validation

| **GAPDH-FW** | CTTTTGCGTCGCCAG |
| --- | --- |
| **GAPDH-REV** | TTGATGGCAACAATATCCAC |
| **DRD2-FW** | CCCTATGGCTTGAAGAGCCTG |
| **DRD2-REV** | TTCAGTGGATCCATCAGGGC |
| **SLIT1-FW** | GTCTGGATGGCTTGAGGACC |
| **SLIT1-REV** | TTGTTCCGCAGCATTAGGGT |
| **LINGO-FW** | CGATTGGTGACCGAGCCG |
| **LINGO-REV** | ATCCTCTTGCTCACCTGCATC |
| **HMCN1-FW** | ACTTTGCGTTGGTGCCTTTC |
| **HMCN1-REV** | TCACTGGGCCAATTTCTGGA |
| **NRP1-FW** | AGACGGGACCCATTCAGGAT |
| **NRP1-REV** | AGTTGCCATCTCCTGTGTGA |
| **SYNAPSIN-FW** | CAGCTCAACAAATCCCAGTCTC |
| **SYNAPSIN-REV** | GGTCTCAGCTTTCACCTCGT |
| **SNCA-AS1-FW** | GAGCGGGCAGACAGATTTTA |
| **SNCA-AS1-REV** | TTTGGAAATCCTGGAGAACG |
| **SNCA-FW** | TGCTGCTGAGAAAACCAAAC |
| **SNCA-REV** | GAAGCACCGAAATGCTGAGT |
